# Improved chilling tolerance in glasshouse-grown potted sweet basil by end-of-production, short-duration supplementary far red light

**DOI:** 10.3389/fpls.2023.1239010

**Published:** 2023-08-16

**Authors:** Firdous U. Begum, George Skinner, Sandra P. Smieszek, Simon Budge, Anthony D. Stead, Paul F. Devlin

**Affiliations:** ^1^ Department of Biological Sciences, Royal Holloway University of London, Egham, United Kingdom; ^2^ Vitacress Herbs, Chichester, United Kingdom

**Keywords:** sweet basil, cold tolerance, end-of-production, phytochrome, light supplementation, soluble sugars

## Abstract

Sweet basil is a popular culinary herb used in many cuisines around the world and is widely grown commercially for retail as a live potted plant. However, basil is easily damaged by temperatures below 12 °C meaning plants must be transported from the grower to the retailer in a warm transport chain, adding considerable commercial cost in temperate countries. Improvement of chilling tolerance has been demonstrated in post-harvest crops such as tomato fruits and, indeed, fresh cut basil, by manipulation of the red:far red ratio of light provided to plants throughout the photoperiod and for a significant duration of the growing process in controlled environment chambers. We tested the effectiveness of periodic short-duration end-of-production supplementary far red light treatments designed for use with basil plants grown in a large scale commercial glasshouse for the live potted basil market. Four days of periodic, midday supplementary far red light given at end of production induced robust tolerance to 24 h of 4 °C cold treatment, resulting in greatly reduced visual damage, and reduced physiological markers of chilling injury including electrolyte leakage and reactive oxygen species accumulation. Antioxidant levels were also maintained at higher levels in live potted basil following this cold treatment. RNAseq-based analysis of gene expression changes associated with this response pointed to increased conversion of starch to soluble raffinose family oligosaccharide sugars; increased biosynthesis of anthocyanins and selected amino acids; inactivation of gibberellin signaling; and reduced expression of fatty acid desaturases, all previously associated with increased chilling tolerance in plants. Our findings offer an efficient, non-invasive approach to induce chilling tolerance in potted basil which is suitable for application in a large-scale commercial glasshouse.

## Introduction

Sweet basil (*Ocimum basilicum* L.) is one of the most popular fresh herbs used throughout the world (Makri and Kintzios, 2008). As well as being a common ingredient in cooking, basil essential oils are commercially extracted for use as flavorings, fragrances, and additives in cosmetics and toiletries (Hiltunen and Holm, 1999). Their chemical composition is rich in linalool and methyl chavicol (Nacar and Tansi, 2000; Marotti et al., 2002). Basil essential oils have also been shown to have strong antimicrobial (Rattanachaikunsopon and Phumkhachorn, 2010) and antioxidant activity (van der Mheen et al., 2010), leading to suggestions of potential uses as a natural preservative (Takwa et al., 2018). As with many culinary herbs, basil is often purchased by consumers as a potted herb, making fresh material directly available from the kitchen windowsill, and this option is becoming increasingly popular. Over the last two decades sales of culinary potted herbs directly to consumers have increased dramatically. In the UK alone, in 2017, the industry produced over 20 million pots for supermarkets and sales of potted basil accounted for 40% of this market (Moncrieff, 2017). Basil is native to tropical and sub-tropical regions. As such, it is sensitive to chilling and temperatures below 12 °C cause strong wilting, discoloration of the leaves and loss of aroma (Fratianni et al., 2017). Fresh basil must, therefore, be maintained above this temperature in order to maintain commercial salability and this presents particular difficulties during distribution of fresh potted basil during winter months. Further issues for suppliers are created by the fact that the warm supply chain required for potted basil is counterproductive for more-temperate herbs such as coriander and parsley where shelf life is increased by maintenance at chilled temperatures (Cantwell and Reid, 1993; Loaiza and Cantwell, 1997). Potted herbs such as coriander and parsley are commonly transported alongside basil in mixed shipments meaning these ideal chilled temperatures cannot be used.

Post-harvest chilling damage during distribution and storage is a problem for a number of tropical and subtropical crops grown in greenhouses in countries with a temperate climate in the same way that it is for potted basil. Changes to membrane lipids resulting in loss of membrane integrity and increases in levels of reactive oxygen species in response to low temperature are two of the key primary effects leading to spoilage at chilling temperatures in many such cases (Sevillano et al., 2009). While temperate crops have mechanisms to adapt to low temperatures to prevent such damage, many tropical and subtropical crops lack the capability to adapt to the degree required and in time for protection mechanisms to be effective (Sevillano et al., 2009). Several studies have examined the induction of cold tolerance in fresh cut basil with a view to extending its shelf life by allowing it to be cold-stored. These treatments include pre- and post-harvest chilling or heat treatments. Conditioning plants using chilling temperature treatments prior to harvest has been shown to reduce damage. Specifically, it was demonstrated that a treatment of just 4 hours at 10 °C for 2 days prior to harvest would considerably extend shelf life of fresh cut basil stored at 5 °C but only if that 4 hour treatment was given at the end of the day (Lange and Camero, 1997). Conversely, Aharoni et al. (2010) demonstrated that exposure of freshly-cut basil shoots to 8 hours of humidified air at 38 oC, immediately prior to cold storage, dramatically reduced subsequent chilling damage. However, the same study suggested that this effect was due to it killing off spores of the spoilage fungus, *Botrytis cinerea* rather than any effect on the plant (Sharabani et al., 1999; Aharoni et al., 2010). Recently pre-harvest abscisic acid (ABA) application has also been found to reduce subsequent post-harvest chilling damage in fresh-cut basil (Satpute et al., 2019).

However, none of these treatments provide a viable approach for potted basil. While heat or cold treatment is viable for cut basil, large scale heat or cold treatments of potted basil would be expensive and technically demanding for the thousands of pots per day commonly supplied by growers. Spraying with ABA for either potted or cut basil is unlikely to be accepted with supermarkets. One study in tomato, however, provided an interesting additional possibility which could provide a very feasible approach to this problem. Wang et al. (2016) demonstrated that the light environment could have a significant impact on cold tolerance in seedlings of tomato. Plants have a wide array of photoreceptors that convey information about the light environment and allow plants to adapt their growth accordingly. Such photoreceptors control physiological and developmental process throughout the life history of the plant (Paik and Huq, 2019). The phytochrome family of photoreceptors detect red and far red wavelengths. They exist in two photo-interconvertible forms: an inactive, red-absorbing Pr form and an active, far red-absorbing Pfr form. Absorption of light converts Pr to Pfr and vice versa. This unique property of the phytochromes enables them to sense the reduction in the red: far red ratio (R:FR) in ambient light associated with vegetative shading. Light reflected from neighboring plants is specifically depleted in red light as a result of chlorophyll absorption resulting in a dramatic reduction in R:FR. This consequently leads to a depletion of the active Pfr form of phytochrome which triggers the shade avoidance syndrome, a response which includes a channeling of resources towards promotion of elongation growth (Franklin and Quail, 2010). Reduced R:FR has been associated with a wide range of changes in resilience in plants (Yang et al., 2016), not least, with increased freezing tolerance in Arabidopsis (Franklin and Whitelam, 2007). Following this observation, Wang et al. (2016) grew tomato plants in an enclosed controlled environment in monochromatic red light (R); monochromatic far red light (FR); or a mixture of monochromatic R and monochromatic FR in various proportions to generate different R:FR ratios throughout their 12 h photoperiod. They found that low R:FR ratios induced cold tolerance in tomato seedlings via mechanisms involving ABA and jasmonate (JA) signaling and increased expression of ABA and JA-regulated genes as well as induction of the C-REPEAT BINDING FACTOR (CBF) stress signaling pathway genes, which have previously been demonstrated to regulate cold resilience in plants (Thomashow, 2010). In a similar experiment, Larsen et al. (2023) very recently also found improved post-harvest chilling tolerance in fresh cut leaves from basil that had been grown in reduced R:FR ratio light throughout an 18 h photoperiod for one or three weeks prior to harvest in a vertical farming system. Larsen et al. (2023), however, found that ABA and JA were not altered in development of low R:FR-induced chilling tolerance in basil; though, they observed increased levels of soluble sugars which they proposed may account for the improved chilling tolerance.

While such FR supplementation is manageable in a small-scale enclosed environment, the use of supplementary far red LED illumination throughout the photoperiod for one to three weeks in large scale, commercial glasshouse production would require considerable investment in further artificial lighting in order to illuminate a large proportion of the production area. Long term supplementary illumination would also add considerable extra energy costs. In addition, supplementary FR, generating R:FR ratios below 1.0 over such a period of time would cause considerable elongation growth in the crop, resulting in “leggy” plants. In a previous study, Larsen et al. (2020) showed that identical low R:FR ratio treatments to those which they later used to induce chilling tolerance also caused close to a 50% increase in plant height after three weeks of treatment. Reduction in leaf area was also seen following one week’s treatment. Unlike fresh-cut basil, such changes in morphology toward more “leggy” plants would not be a welcome trade-off for growers of potted basil, with retailers and customers associating a uniform, dense growth with healthy plants. Taller plants could also have the drawback of rendering pots unsuitable for current packaging.

Work by Fowler et al. (2005) demonstrated that induction of the cold response pathway in Arabidopsis, which confers freezing tolerance, is gated by the circadian clock, with the midday period being crucial for induction by cold treatment. Arabidopsis is tolerant of cold but requires a physiological priming, usually with cold treatment, in order to become tolerant of mild freezing conditions. Franklin and Whitelam (2007) demonstrated that treatment with low R:FR light can also trigger freezing tolerance in Arabidopsis. They found that simulated shade treatment upregulated the common CBF cold/drought responsive factor genes, indicating another example of crosstalk among stress responsive pathways. Building upon the work of Fowler et al. (2005); Franklin and Whitelam (2007) also demonstrated the key involvement of the circadian clock in gating the induction of the cold response pathway in Arabidopsis, meaning that mid-day FR supplementation alone was sufficient and induced the strongest response. They demonstrated that 4 h supplementary FR treatment around midday over a 4 day period was sufficient to induce freezing tolerance. Crucially, such mid-day 4h FR treatment would not induce significant shade avoidance phenotype (Cole et al., 2011).

Consequently, we sought to examine whether chilling tolerance could be induced in potted basil by 4 h supplementary FR treatment around midday. Also following the protocol of Franklin and Whitelam (2007), we sought to test whether 4 days of treatment at end of production was sufficient to induce this. In testing potted basil as opposed to fresh-cut basil, we also sought to examine whether the benefit of this supplementary FR could be maintained in living plants for sufficient duration to encompass transport to the retailer. We also sought to examine the effect of this four days of end-of-production, periodic midday supplementary FR on the transcriptome in order to better understand the impact at the molecular level.

We demonstrated that 4 h mid-day supplementary FR given over the final four days at end of production was able to confer greatly-improved chilling tolerance in potted sweet basil. Four hours of mid-day supplementary FR, below the minimal duration known to induce shade-related elongation growth (Cole et al., 2011), was applied periodically at midday, the time of maximal sensitivity for induction of cold tolerance pathways due to circadian gating (Kidokoro et al., 2009). Visible assessment of chilling injury and biochemical assessment of physiological markers of cellular damage demonstrated that, unlike control plants, FR treated plants sustained very little injury from 24 h incubation at 4 °C. Antioxidant levels, likewise remained high in FR-treated but not control plants following chilling. Transcriptomic analysis revealed changes in gene expression associated with a number of biochemical pathways previously linked with chilling tolerance. Increased expression of genes encoding enzymes involved in mobilization of starch into soluble sugars, particularly, raffinose and stachyose; synthesis of protective anthocyanins; and synthesis of amino acids was observed. We, therefore, demonstrate that basil’s tolerance towards chilling temperatures can be increased through changes in growing conditions that could feasibly be applied in a commercial glasshouse setting; thus, improving shelf life and reducing wastage, while also providing understanding of the impact of such changes within the plant at the molecular level.

## Materials and methods

### Plant material and growth conditions

Pot-grown sweet basil, *Ocimum basilicum* var. Marian (Enza Zaden, UK), were grown by Vitacress Herbs (Runcton, UK) in a commercial glasshouse. All pots used in this assay were collected in winter at 50 days old at which point they were ready for the market. Plants were grown in TPS peat substrate mix (Jiffy Products International, Moerdijk, The Netherlands). This contained 1 kg m^-3^ Tref Base fertilizer (final NPK+Mg: 17:10:14 + 4 plus trace elements, pH 5.8) and 0.25 ml m^-3^ FIBA-ZORB water holding agent (Turftech, Preston, UK) with 30 seeds per 0.4 L pot. Pots were maintained in a tightly packed arrangement in darkness for the first seven days. Pots were then spaced to 70 pots m^-2^ and the plants were grown under natural light, supplemented as necessary over a central 12 h period during the day by SON-T high pressure sodium lamps (140 μmol m^-2^ s^-1^ photosynthetically active radiation, PAR) when the natural light was less than 180 μmol m^-2^ s^-1^ PAR. This results in a photoperiod of 12 h in winter, stretching up to 18 h in summer. Average daylight PAR at the growing bench peaks at approximately 85 μmol m^-2^ s^-1^ at midday in winter up to 230 μmol m^-2^ s^-1^ at midday in summer, with additional shading is applied to the glasshouse in summer. Typical daily light integral (DLI) ranges from 8 mol m^-2^ d^-1^ in winter up to 12 mol m^-2^ d^-1^ in summer and this corresponds to a typical growth period of up to 52 days in winter, and approximately 32 days in summer. Plants were grown under controlled ambient conditions with an average temperature of 20.2 °C ( ± 0.8 °C) during the day and 18.3 °C ( ± 0.9) during the night. During the first 7 d, pots were watered with potable water as required to keep the substrate moist. Watering was carried out from the base, as required, by flooding pots 20 minutes at a depth of 3 cm then draining. After spacing they were then watered with water and/or Tref Base fertilizer in order to keep the substrate moist while maintaining electrical conductivity between 2.2 to 2.8 μS cm^-1^.

Pots were transferred from the Vitacress glasshouse to our experimental facility at the end of the 12 h photoperiod. Before transfer, pots were watered by flooding with potable water for 15 min. They were then placed in darkness for 12 h at 21 °C before transfer to the following experimental conditions. Control plants were maintained in 12 h white light/12 h dark cycles at 21 °C for four days. This provided a PAR of 50 μmol m^-2^ s^-1^ and a R:FR ratio of 5.11 (the ratio of intensity of 10 nm bandwidths centered around 660 nm and 730 nm). Experimental plants were maintained in the same 12 h white light/12 h dark conditions but were additionally treated with mid-day supplementary FR illumination from 4 h after dawn to 8 h after dawn, generating a R:FR ratio of 0.16. White light was provided by Osram Lumilux T5 HE 35 W/830 cool-white fluorescent tubes. FR illumination was provided by FR LEDs (Λ max 735 nm, Shinkoh Electronics, Tokyo, Japan). All light measurements were made using a StellarNet EPP2000-HR spectroradiometer (StellarNet Inc., Tampa, FL, USA). After 4 d, plants were transferred at 4 h after dawn to either 21 °C or 4 °C for 24 h. This final incubation was carried out in dim light conditions (5 μmol m^-2^ s^-1^, Osram Lumilux T5 HE 35 W/830 cool-white fluorescent tubes) to simulate typical lighting conditions during commercial transport and storage.

### Visual assessment of chilling damage

A scale for the grading of the basil pots exposed to chilling temperature was based on preliminary observations which demonstrated that chilling damage was first observed in the form of leaf wilting followed by discoloration of the leaves. A scale ranging from 1 to 5 was devised, where 1 represented no visible damage; 2 was scored if some plants within a pot exhibited only slightly wilted leaves; 3 was scored where some plants within a pot exhibited heavily wilted leaves; 4 was scored if some plants within a pot exhibited wilted leaves and discoloration was observed on less than 50% of the leaves in the pot; and 5 scored if some plants within a pot exhibited wilted leaves and discoloration was observed on more than 50% of the leaves in the pot. Representative pictures of pots exhibiting each level in the grading scale are shown in **Supplementary Figure 1**.

### Measurement of electrolyte leakage

The electrolyte leakage assay was modified from the methods described by Campos et al. (2003) and Bajji et al. (2002). At the end of the environmental treatment period, the six top-most fully expanded leaves from the basil plants in a single pot were removed to form each replicate and immediately immersed in 100 ml of distilled water in a crystallizing dish (150 ml). The leaves within the crystallizing dish were separated from one another by a nylon mesh. The crystallizing dishes were then incubated at 21 °C in darkness on an orbital shaker at 40 RPM. The electrical conductivity (EC) of the water in microSiemens (μS) was measured to obtain initial EC (ECi) and EC of the water containing leaf samples was measured after 12 h of incubation to obtain a final EC (ECf). The water and leaves was then autoclaved at 120 °C and 15 psi for 30 min, and another EC reading was taken to obtain total EC (ECt). Relative electrolyte leakage (EL) was calculated using the formula EL (%) = (ECf - ECi)/(ECt - ECi) x 100.

### DAB (3,3′-diaminobenzidine) staining

The DAB assay was carried out according to the protocol described by Daudi and O’Brien (2012). To prepare the DAB staining solution, 0.1% DAB was prepared with sterile H_2_O, with the pH being reduced to 3.0 with 1M HCl to allow dissolution of the DAB. Tween 20 was then added to 0.05% (v/v) and Na_2_HPO_4_ to a final concentration of 10 mM. The three top-most fully expanded basil leaves per replicate pot were removed and placed within a sterile Petri dish then immersed in 25 ml of the DAB staining solution. Leaves inside the Petri dishes were vacuum infiltrated using a gentle vacuum for 5 min then the dishes were covered with aluminum foil and incubated at 30 °C for 4-5 h on an orbital shaker at 55 RPM. Following incubation, the DAB staining solution was removed and replaced by a bleaching solution (ethanol: acetic acid: glycerol = 3:1:1) in order to bleach chlorophyll. The plates were incubated for 15 min at 95 °C then the bleaching solution was removed and replaced by fresh bleaching solution before incubation for a further 30 min at 95 °C. Following this, the leaves were rinsed with water and then photographed under uniform lighting. Relative H_2_O_2_ accumulation was calculated using ImageJ (Schneider et al., 2012), using the color threshold tool to measure the area of a leaf covered by the brown precipitate and expressing this as a percentage of the area of the entire leaf.

### FRAP assay

Water soluble antioxidants were assayed using the ferric reducing ability of plasma (FRAP) assay as described by Benzie and Strain (1996). The top-most fully expanded single leaves were weighed then flash frozen in liquid nitrogen in 1.5 ml microcentrifuge tubes and stored at -80 °C until assayed. For the FRAP assay, these leaves were ground to a fine powder in a pestle and mortar with sand. After grinding, 0.6 ml of acetate buffer (300 mM, pH 7.6) was added to the mortar and the lysate was then transferred to a new 1.5 ml microcentrifuge tube. A further 0.6 ml of acetate buffer was added to the mortar to recover the remaining lysate which was also added to the same microcentrifuge tube. The lysate was then centrifuged at 15,000 g for 4 min and the supernatant, containing the water-soluble antioxidants, was transferred into a clean 1.5 ml microcentrifuge tube.

Antioxidant activity was compared to that of ascorbic acid standards using freshly prepared FRAP solution (25 ml of 300 mM acetate buffer pH 3.6, 2.5 ml of 20 mM Ferric chloride hexahydrate and 2.5 ml of 10 mM 2,4,6-Tripyridyl-s-Triazine in 40 mM HCl). To 30 μl of the sample or standard, 300 μl of FRAP solution was added. The intensity of the colored product resulting from reduction of Fe^3+^ ions to Fe^2+^ was determined by measuring absorbance at 590 nm using a SpectraMax Plus 384 microtiter plate spectrophotometer (Molecular Devices, Wokingham, UK). Ascorbic acid standard curves with correlation coefficients of not less than 0.99 were used to quantify the antioxidant activity in the experimental samples and antioxidant activities were then normalized by fresh weight.

### Extraction of total RNA from basil tissues

Total RNA was extracted from leaf tissues of basil using an RNAeasy plant mini kit (Qiagen, Manchester, UK). Total RNA was extracted from the top-most fully-expanded single leaves from at least five biological replicates for each environmental condition and was pooled into one sample for each condition. RNA integrity and purity were checked by carrying out RNA gel electrophoresis, and by measuring the absorbance at 260 nm, 280 nm and 230 nm using a Nanodrop spectrophotometer ND-1000 (Qiagen, Manchester, UK).

### RNA sequencing

All samples were sequenced at the sequencing core facility, Cleveland Clinic, Cleveland Ohio. Prior to sequencing, sample quantity and quality was measured using a Qubit fluorometer (Thermo Fisher Scientific – Waltham, MA, USA). The RNA sequencing library was prepared using the protocol described by (Wang et al., 2011). Briefly, mRNA was obtained from the total RNA pool before fragmentation, cDNA synthesis, adapter ligation and size selection; and the size-selected ligated DNA products were then amplified by PCR to produce a sequence-ready library. The cDNA was then subjected to paired-end high-throughput sequencing using an Illumina Hiseq2500 (San Diego, CA, USA).

### Differential expression analysis

Analysis of RNAseq data was carried out via the Galaxy platform (Afgan et al., 2022). Quality of raw sequence data was assessed through the FastQC package (Andrews, 2010). Adapter sequences were removed with the Cutadapt package (Martin, 2011) and sequences with a low-quality score were processed with the Trimmomatic package using default settings (Bolger et al., 2014). A *De Novo* transcriptome assembly was constructed using the Trinity 2.2.0 package (Haas et al., 2013) with paired reads used as input. Forward and reverse read trimmed sequence files were pairwise aligned to the Trinity assembled transcripts using the HISAT2 2.1.0 package (Kim et al., 2015). Aligned reads were then assembled using StringTie 1.3.4 (Pertea et al., 2015). The StringTie assembled transcripts for the various samples were then combined using StringTie merge 1.3.4 (Pertea et al., 2015) into a single non-redundant list that was used as a global set for reference for differential expression analysis between the samples. GFFCompare 0.9.8 (Pertea et al., 2020) was then used to compare the StringTie merge global set to the Trinity assembled transcripts in order to annotate transcripts with respective Trinity ID codes. Gene expression was then measured using the featureCounts 1.6.3 package (Liao et al., 2014). Annotated transcripts from GFFCompare were input with aligned transcripts from the HISAT2 package to output a table containing counted fragments per gene. Data from featureCounts was then used to produce a table of normalized FPKM counts for the comparison of gene expression across the tested samples.

In order to carry out functional analysis, the Trinity assembly file was annotated with gene identifiers using the NCBI BLAST+ BLASTx command line application, using a reference database created from the Uniprot Arabidopsis proteome (uniprot.org, ID: UP000006548). Arabidopsis Genome Initiative (AGI) TAIR10 ID annotations were applied where the p-value of the top match was below 1x10^-4^ and the bit score above 50. Where multiple fragments were mapped to the same AGI ID, their expression was averaged per AGI ID. The Panther functional classification tool (Mi et al., 2019) was used to assign transcriptome-wide biological process GO tags.

For relative expression analysis a minimum expression cut-off of 15 on average across the four samples for each AGI ID was first applied to the data. Relative change in expression for each AGI ID for the comparison between control and FR treated samples at each timepoint was then expressed as a log base 2 value. The PageMan 0.12 utility (Usadel et al., 2006) using AGI TAIR10 mappings was then used to assess overrepresentation of biological process gene ontology categories among differentially expressed genes, applying the default parameters. Z-scores for over- or under-represented biological processes were then represented using the conditional formatting function in Microsoft Excel. Highly differentially expressed genes were also assessed for over-representation of GO-Slim Biological Process tags via the Panther enrichment analysis tool using the Fisher’s Exact test (Mi et al., 2019).

### qRT-PCR

Reverse transcription of extracted RNA was performed with the QuantiTect Reverse Transcription Kit (Qiagen, Manchester, UK). gDNA was removed as per instructions, then 1 μg of RNA per sample was reverse transcribed. The cDNA synthesis was performed in a Techne 5-prime thermocycler (Cole-Parmer Ltd., Saint Neots, UK).

The primers used for assessing expression gene by qPCR were designed based on the Trinity-assembled transcripts, with the exception of *O. basilicum* Ubiquitin primers which were designed based on a published sequence obtained from GenBank: LN999820.1. The primer pairs were designed (150 bp maximum product length, optimal Tm at 60°C, GC % between 45 and 65%) using Primer3 software (Untergasser et al., 2012), favoring the 3’ end of transcript sequences. Primer sequences are shown in **Supplementary Table 1**. In order to determine the specificity for the target sequence, standard PCR from cDNA was first carried out followed by sequencing of the product. PCR was performed using PCR BioMix™ Red PCR reaction mixture (Bioline, London, UK). To confirm that the PCR was successful, 5 µl of each PCR product was run on a 3% TBE (Tris-borate-EDTA) agarose gel alongside a Biolone HyperLadder™ 50 bp DNA ladder (Bioline, London, UK) to check for bands of the expected size. To be certain the primers were amplifying the target genes of interest, the gel-purified PCR products were sent to Eurofins Genomics (Wolverhampton, UK) for DNA sequencing. 25 µL of PCR product was run on a 2% TBE agarose gel. The bands were then cut out and purified as per the Wizard^®^ SV Gel and PCR Clean-Up System (Promega, Southampton, UK) centrifugation protocol. 2 µl of loading dye (Bioline, London, UK) was mixed with 3 µl of the DNA extracted from the clean-up and run on a 2% TBE agarose gel to check the band intensities for DNA concentration. The cleaned DNA was then diluted and mixed with the necessary primers as per the Eurofins Genomics Mix2Seq protocol.

qPCR was performed in a Rotorgene 6000 thermocycler (Qiagen, Manchester, UK) using the primer sequences shown in **Supplementary Table 1**. *O. basilicum* Ubiquitin was used as the housekeeping gene. Three biological replicates and two technical replicates were used for each treatment sample. Samples were prepared with 0.5 μg of cDNA in a total volume of 20 µl, using the QuantiTect SYBR Green PCR Kit (Qiagen, Manchester, UK) with each primer added to a final concentration of 200 nM. A QIAgility automated pipetting robot (Qiagen, Manchester, UK) was used to prepare reactions in order to maximize pipetting accuracy. The following temperature program was used for qPCR: an initial denaturing of 94 °C for 2 min, followed by a cycle of 94 °C for 15 s, 60 °C for 45 s and 72 °C s for 30 cycles. Following qRT-PCR, melting curves for the products were obtained using the Rotor-Gene Q (ver.2.3.1.49) software (Qiagen, Manchester, UK) in order to confirm amplification of a single target. Ct values for the qPCR data were then obtained using the Rotor-Gene Q software and used to calculate relative expression using the ΔΔCt method (Rao et al., 2013).

## Results

### Daily, mid-day supplementary FR at end of production reduces appearance of chilling injury in greenhouse-grown potted basil

**Figure 1:**
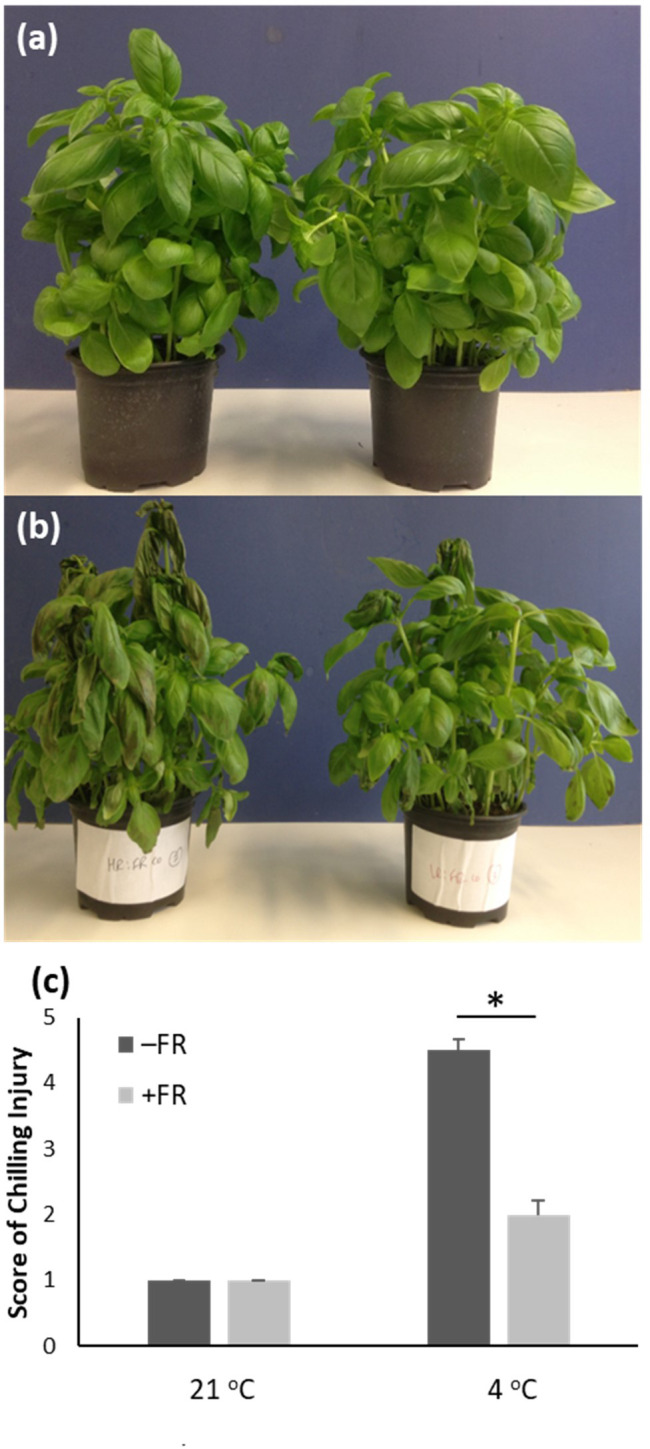
End-of-production midday supplementary FR treatment reduces chilling injury in potted basil. Glasshouse-grown market-ready basil plants were maintained at 21 °C in 12 hr light/12 h dark cycles either with 4 h supplementary FR in the middle of the day (right in images a and b) or without supplementary FR (left in images a and b) for 4 days. Plants were then either maintained in 21 °C **(A)** or transferred to 4 °C for 24 h **(B)**. Images show representative pots. **(C)** Mean chilling injury score based on phenotypic assessment for plants treated as above. Values represent mean ± SE for a minimum of seven pots. Asterisk represents significant difference, p ≤ 0.05.

Greenhouse-grown potted sweet basil plants at a stage ready for market were transferred at dusk to a controlled environment undergoing 12 h white light: 12 h dark cycles at 21 °C. They were then either maintained in these control conditions or given a daily treatment of 4 h supplementary FR, generating a R:FR ratio of 0.16, beginning four hours (4 h) after dawn and spanning the middle of the day for four consecutive days. Plants from each treatment group were then either transferred to dim light at 21 °C or to dim light at 4 °C for 24 h to simulate low temperature transport and storage conditions. Damage due to chilling was then scored using a range of metrics. All plants which had been transferred to chilling conditions showed some degree of chilling injury, exhibited as wilting and leaf discoloration (**Figures 1A, B**). However, plants which had received supplementary FR treatment prior to transfer showed greatly reduced visible chilling injury following 4 °C treatment compared to untreated plants. While the majority of untreated plants showed both wilting and extensive discoloration, the majority of FR-treated plants within each pot showed no wilting and only minor discoloration (**Figures 1A, B**). Quantification of chilling injury using a scale assessing both the occurrence of wilting and the degree of discoloration, revealed a significant difference between control and FR-treated plants following cold treatment (**Figure 1C**). FR treatment, however, caused no visible difference in plants maintained in warm 21 °C conditions, notably confirming that, as expected, the plants showed no elongation growth or other morphological shade avoidance response to the mid-day FR treatment.

### Markers of cellular damage and stress reveal a strong cold-protective effect of FR treatment

**Figure 2:**
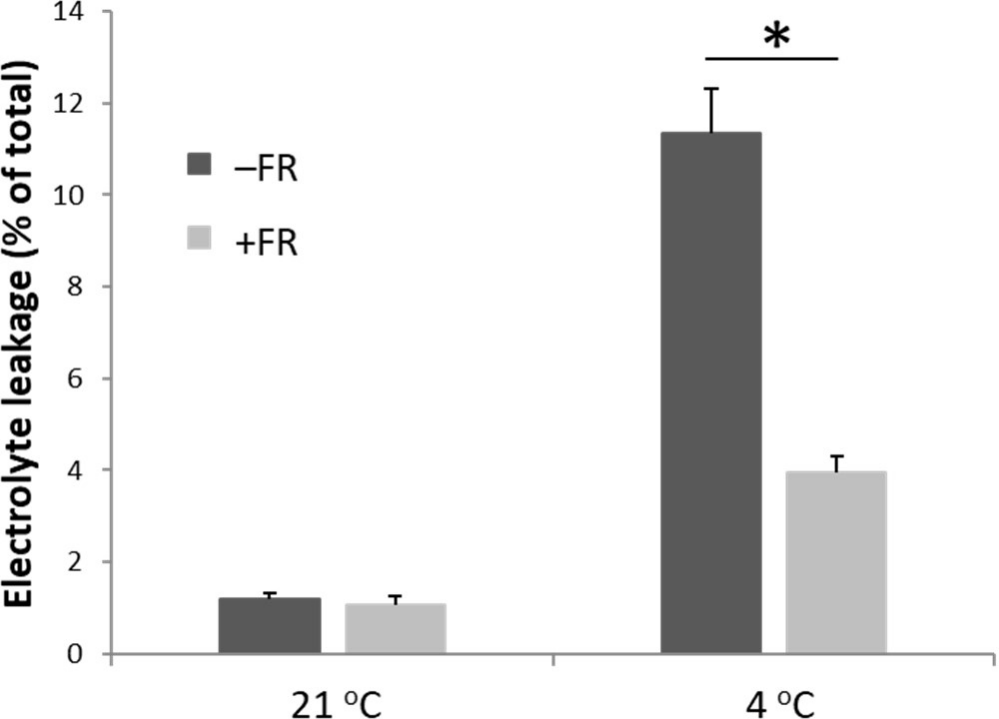
End-of-production midday supplementary FR pre-treatment reduces electrolyte leakage from leaves following chilling. Electrolyte leakage from leaves was measured in leaves of basil either maintained in control conditions or treated with 4 h midday supplementary FR for four days at end of production. Plants were then either maintained in 21 °C or transferred to 4 °C for 24 h prior to measurement. Data are mean ± SE for a minimum of 10 plants. Asterisk represents significant difference, p ≤ 0.05.

Electrolyte leakage from leaves was used as a proxy for cellular damage following cold treatment. Only minimal electrolyte leakage was observed in leaves from plants maintained in warm conditions and daily mid-day FR treatment had no effect on this (**Figure 2**). Cold treatment resulted in a significant increase in electrolyte leakage in leaves of both control and FR treated plants. However, levels of electrolyte leakage following cold were greatly reduced in plants that had previously been treated with FR compared to plants that had previously been maintained in control conditions (**Figure 2**). Thus, FR pre-treatment appeared to enable the plants to withstand much of the damaging effect of cold on the membranes of leaf cells.

**Figure 3:**
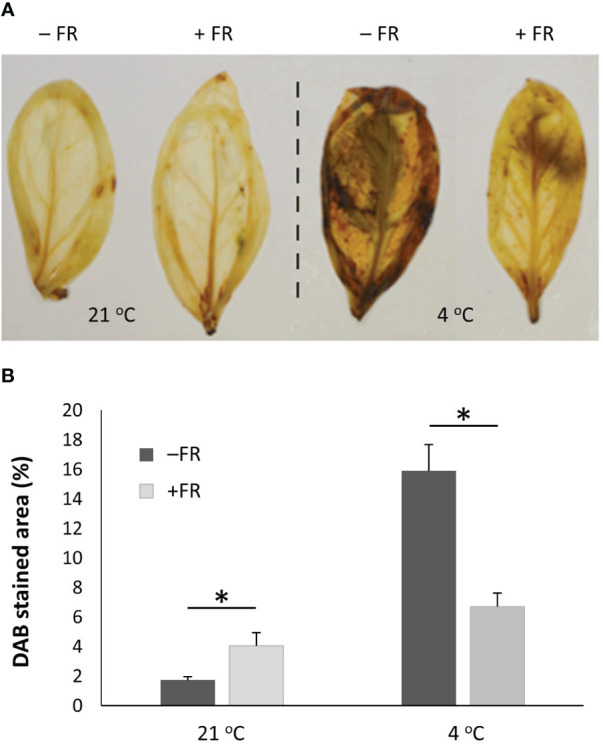
End-of-production midday supplementary FR pre-treatment reduces ROS accumulation in leaves following chilling. Plants were either maintained in control conditions or treated with 4 h midday supplementary FR for four days at end of production. Plants were then either maintained in 21 °C or transferred to 4 °C for 24 h **(A)** Representative leaves from a subsequent DAB staining assay revealing accumulation of hydrogen peroxide in basil leaves. **(B)** Percentage of leaf area showing DAB staining. Data are mean ± SE for a minimum of 15 leaves. Asterisks represent significant differences, p ≤ 0.05.

Colorimetric assessment of Reactive Oxygen Species (ROS) accumulation was also carried out as a marker of plant stress at the cellular level. A DAB (3,3′-Diaminobenzidine) assay of whole leaves for the detection of H_2_O_2_ revealed that the leaves of plants which had been incubated at 4 °C contained significantly higher levels of H_2_O_2_ than those maintained in 21 °C. However, while high H_2_O_2_ levels were observed in both control and FR-treated basil leaves following cold, leaves of plants previously treated with 4 h mid-day supplementary FR for four days displayed much lower levels of H_2_O_2_ accumulation than those of untreated plants (**Figure 3A**). Quantification of percentage of leaf area revealing H_2_O_2_ accumulation demonstrated that FR-treated plants showed 58% lower H_2_O_2_ levels than untreated plants following cold exposure, indicating a significant difference in the degree of stress between FR-treated and untreated plants. Curiously, however, DAB staining did also reveal a small but significant increase in H_2_O_2_ accumulation as a result of the daily FR pulses in plants maintained in warm 21 °C temperature (**Figure 3B**) suggesting that this treatment did trigger a mild stress response in itself.

Crucially, though, the above assays for cellular damage and redox stress both confirmed a cold-protective effect of daily, mid-day supplementary FR.

### Midday supplementary FR treatment reduces loss of antioxidants following chilling

**Figure 4:**
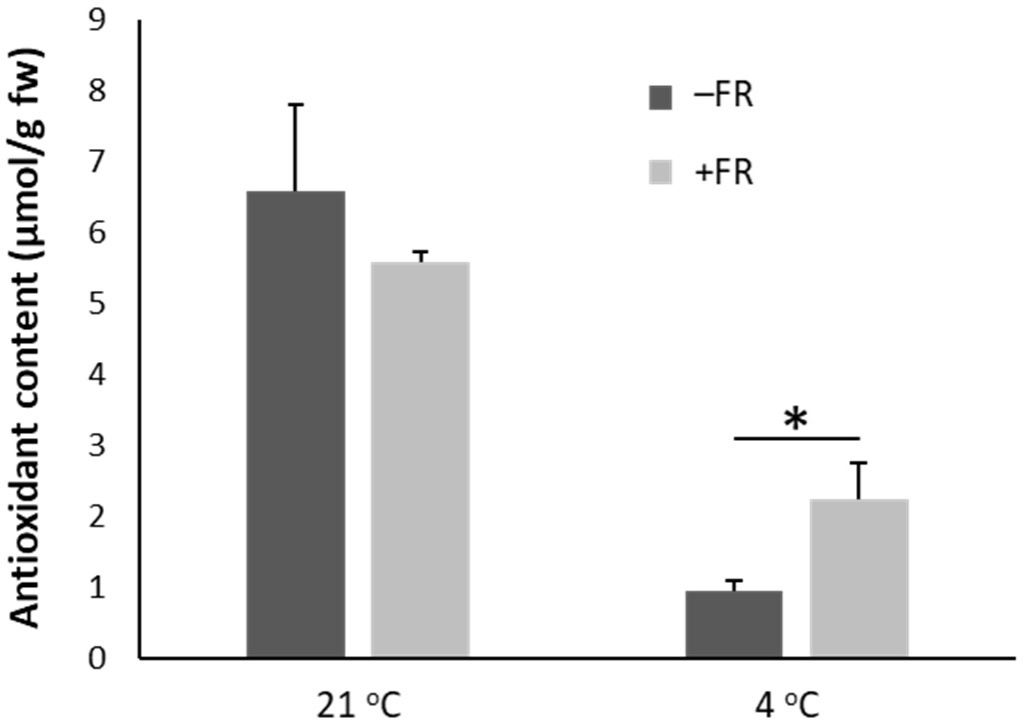
End-of-production midday supplementary FR pre-treatment reduces loss of antioxidants in leaves following chilling. Content of water-soluble antioxidants (equivalent to ascorbic acid, µmol/g fresh weight of leaf material) in leaves of basil either maintained in control conditions or treated with 4 h midday supplementary FR for four days at end of production. Plants were then either maintained in 21 °C or transferred to 4 °C for 24 h. Data are mean ± SE for a minimum of 10 plants. Asterisk represents significant difference, p ≤ 0.05.

A FRAP assay measuring water soluble antioxidant content revealed that chilling resulted in a strong reduction in antioxidant content in basil plants. Although the FR treatment did not prevent a reduction in antioxidant content, the reduction was much less in FR treated plants than in control (**Figure 4**). Following cold treatment, water soluble antioxidant levels in control plants fell to 15% of those observed in plants maintained in warm conditions (an 85% reduction) whereas antioxidant levels in FR pre-treated plants fell to 40% (a 60% reduction). Thus, in addition to reducing damage at both a whole plant and cellular level, daily mid-day FR pre-treatment also helped maintain beneficial antioxidant levels in potted basil during chilling.

### Global gene expression analysis reveals extensive transcriptomic reprogramming by midday FR

In order to determine the possible mechanism of action of end-of-production daily mid-day FR treatment in conferring subsequent cold tolerance in potted basil, we carried out RNA sequencing on FR-treated plants. Samples were taken from basil plants at the midpoint of the fourth and final mid-day FR treatment and two hours post treatment in order to examine both acute and sustained changes in gene expression. The R:FR ratio of 0.16 generated by supplementary FR would be expected to trigger changes in gene expression associated with shade avoidance during the treatment (Devlin et al., 2003). However, following removal of FR, the R:FR ratio returned to 5.11, which would immediately return the phytochrome photoequilibrium to that found in an unshaded environment and reverse any changes in directly phytochrome-regulated gene expression (Roig-Villanova et al., 2006), allowing more-prolonged downstream effects to be identified. In the absence of a high-quality complete reference genome for the complex tetraploid genome of sweet basil (Gonda et al., 2020), we used *De Novo* assembly to reconstruct the transcriptome for the purpose of identifying biological processes impacted by mid-day FR treatment. Following *De Novo* assembly of the transcriptome, gene expression levels were estimated by mapping clean sequence fragments to the assembled transcriptome. The abundance of the transcripts was normalized using the FPKM method. In order to allow later gene ontology analysis, the transcripts were then blasted against a reference database created from the Uniprot Arabidopsis proteome in order to map them onto closely related Arabidopsis proteins. Arabidopsis was chosen as a target due to the far more extensive functional designation of its proteome compared to that of basil or more closely-related fully-sequenced species for which little ontological information is available. The Blast-x tool, identified transcripts corresponding to 9,728 different orthologues of Arabidopsis proteins within the basil RNAseq transcriptome (**Supplementary Table 2**).

**Figure 5:**
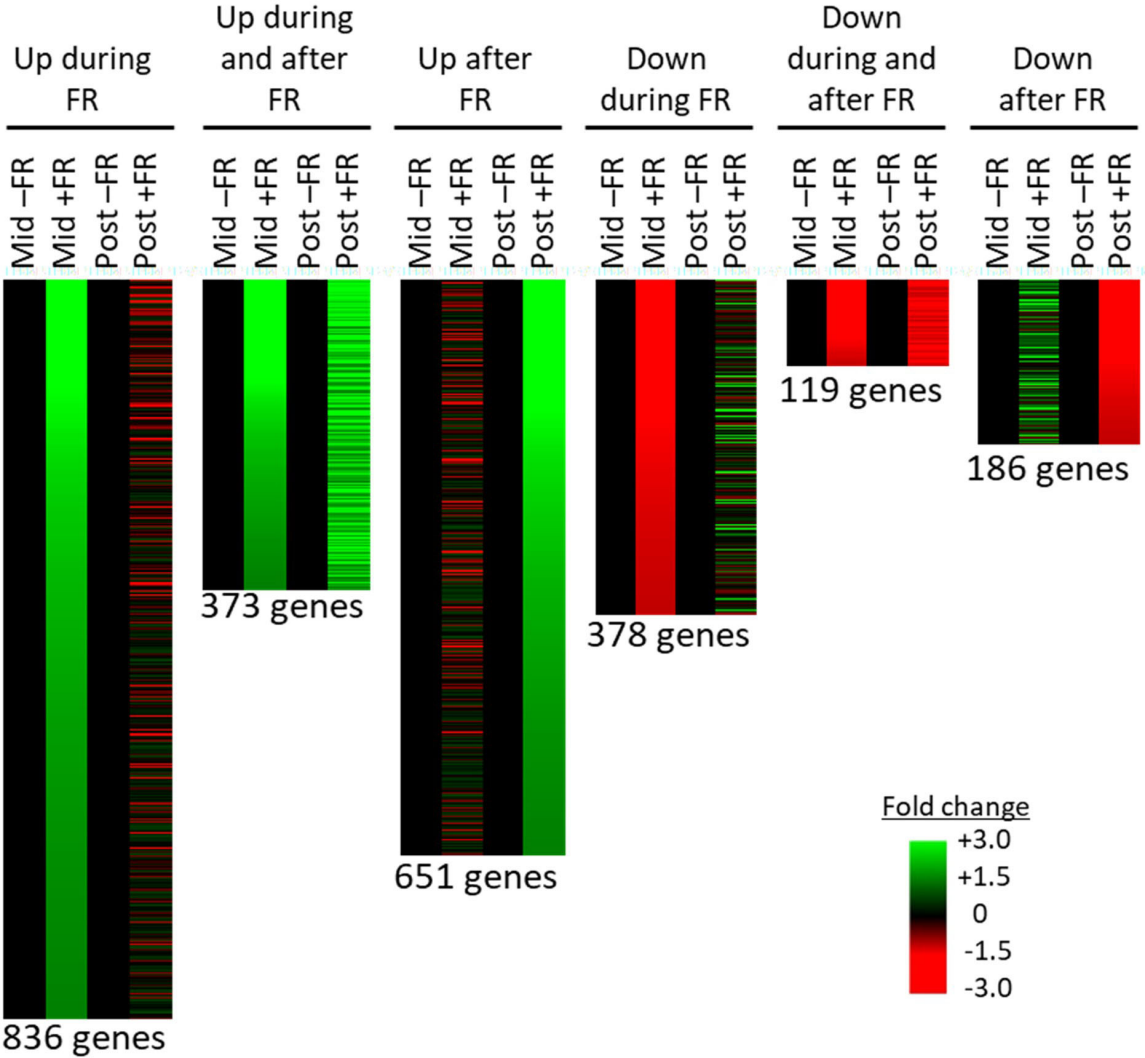
RNAseq reveals persistent changes in gene expression in basil as a result of end-of-production midday supplementary FR treatment. Transcripts identified by RNAseq showing at least two-fold change in abundance during (Mid) and/or 2 h after (Post) midday supplementary FR treatment. Transcripts are grouped according to pattern of response. Each transcript is represented by a separate line and fold change in treated (+FR) versus control plants (–FR) is shown for each time of sampling. Number of transcripts (genes) in each group is shown below each pattern.

A global correlation analysis showed a high degree of correlation between the mapped transcriptomes of control versus FR treated plants; however, a number of genes showed greater than two-fold up or down regulation during or after FR treatment (**Supplementary Figure 2**). Mapped genes showing at least two-fold change in expression either during, or 2 h after, supplementary FR treatment were selected and assigned to groups based on their pattern of their response across the two time points. 671 genes showed at least a two-fold upregulation in expression during but not after FR treatment (no more than a 1.5-fold upregulation after FR treatment). 288 genes showed at least a two-fold upregulation both during and after supplementary FR treatment, while 300 showed at least a two-fold upregulation after supplementary FR but not during FR treatment (**Figure 5**). The lower number of genes showing persistent upregulation is consistent with the removal of the stimulus prior to the second time point; however, it is clear that there is a lasting effect of the treatment. In terms of downregulated genes, 243 genes showed at least a two-fold downregulation in expression in response to FR during but not after the FR treatment (no more than a 1.5-fold downregulation after FR treatment). 40 genes showed at least a two-fold downregulation both during and after supplemental FR, while a further 151 showed at least a two-fold downregulation after supplemental FR with no congruent change during FR treatment (**Figure 5**). Again, although fewer genes showed a persistent pattern of regulation, there is a clear prolonged effect of the FR treatment on a substantial number of genes, consistent with the observed ongoing effect observed for chilling tolerance.

### Periodic midday FR treatment enhances expression of soluble sugar, selected amino acid, and anthocyanin biosynthetic pathways

**Figure 6:**
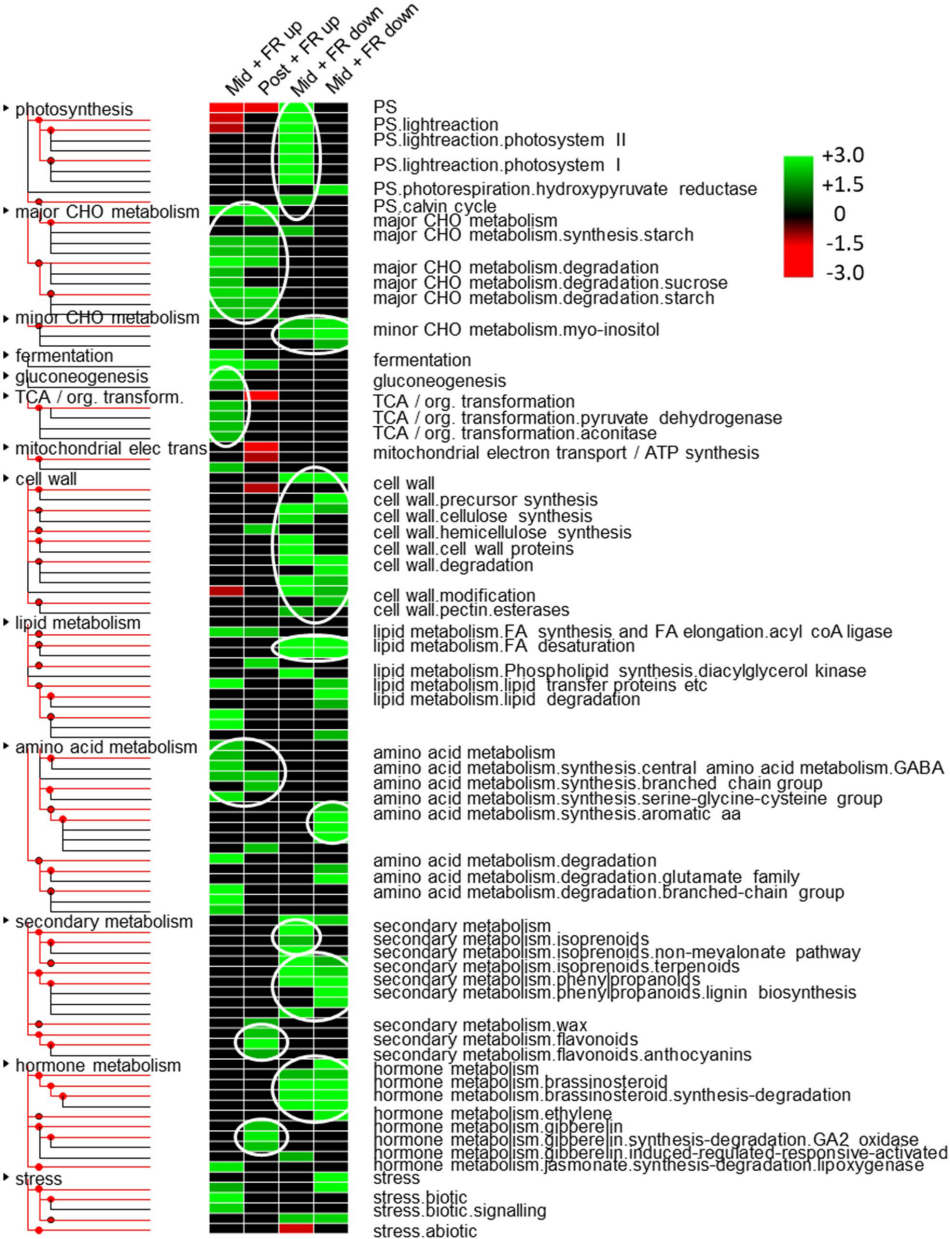
Multiple metabolic, signalling and stress-responsive pathways are affected by end-of-production midday supplementary FR. Over and under-represented MapMan gene ontology categories found among up and down-regulated genes based on pairwise comparisons between control and FR-treated plants during treatment (Mid) and 2 h after treatment (Post). Colored boxes indicate statistically-significant, over-represented categories (p-value below 0.05 based on Fisher’s exact test). The color scale represents z-score, with green indicating gene ontology categories that are over-represented, and red indicating under-represented categories. Text alongside each row provides the MapMan annotation. Non-significant categories are not shown. Tree branches show “parent” and “child” gene ontology terms. White ovals indicate key gene ontology category term branches showing coordinated patterns of enrichment.

In order to examine the likely long term physiological effects on the plant, we next undertook a gene ontology (GO) analysis. An initial genome-wide GO analysis of biological processes based on all mapped transcripts revealed that 79% of these transcripts fell within the parent categories, “cellular process”, “metabolic process”, “biological regulation”, “localization”, “response to stimulus”, and “signaling” (**Supplementary Figure 3**). However, to analyze biological processes enriched among genes identified as up or downregulated during and/or after FR supplementation, an enrichment analysis was then performed using more detailed classifications. A strong enrichment of photosynthesis-related terms was observed among genes temporarily downregulated during FR treatment. This includes genes associated with both photosystems I and II, and the Calvin Cycle. This was complemented by an underrepresentation of genes in this functional category among genes temporarily upregulated during FR. However, in both cases, this effect was no longer observed 2 h after FR treatment had ceased (**Figure 6**). At the same time, there was a strong overrepresentation of genes identified with gluconeogenesis and the TCA cycle among genes temporarily upregulated during but not after FR treatment. Several other enriched parent GO terms were also identified but almost all of those showed enrichment among genes showing persistent differential expression both during and after FR treatment. This includes extensive enrichment of child terms of the major carbohydrate metabolism (major CHO metabolism) parent GO term, particularly starch and sucrose metabolism, with starch metabolism being overrepresented among upregulated genes both during and after FR treatment and sucrose metabolism being overrepresented among upregulated genes during FR treatment. By contrast, myoinositol metabolism was enriched among persistently-downregulated genes. The analysis suggested that cell wall metabolism is downregulated in a persistent way, with terms including both cellulose synthesis and cell wall degradation, cell wall proteins, and pectin esterases enriched among genes downregulated both during and after FR treatment. Similarly, lipid metabolism was indicated as being generally downregulated with fatty acid synthesis and lipid degradation terms being enriched among persistently downregulated genes. Notably, fatty acid desaturation was enriched among genes downregulated following FR supplementation (**Figure 6**). The different branches of amino acid metabolism showed differential enrichment among up versus downregulated genes. Terms associated with branched chain amino acid (BCAA) synthesis, and serine-glycine-cysteine group synthesis were overrepresented among FR upregulated genes while aromatic amino acid synthesis was overrepresented among FR downregulated genes. Likewise, while the analysis suggests a general and long-term downregulation of secondary or specialized metabolism, and particularly of isoprenoid and phenylpropanoid metabolism, there was an enrichment of genes associated with flavonoid and, specifically, anthocyanin metabolism among genes upregulated after FR treatment. Hormone metabolism also showed a pathway-specific enrichment. Brassinosteroid and ethylene metabolism terms were overrepresented among downregulated genes while gibberellin (GA) and jasmonate (JA) metabolism terms were overrepresented among upregulated genes (**Figure 6**).

### qRT-PCR confirms increased expression of genes associated with mobilization of starch into soluble sugars

**Figure 7:**
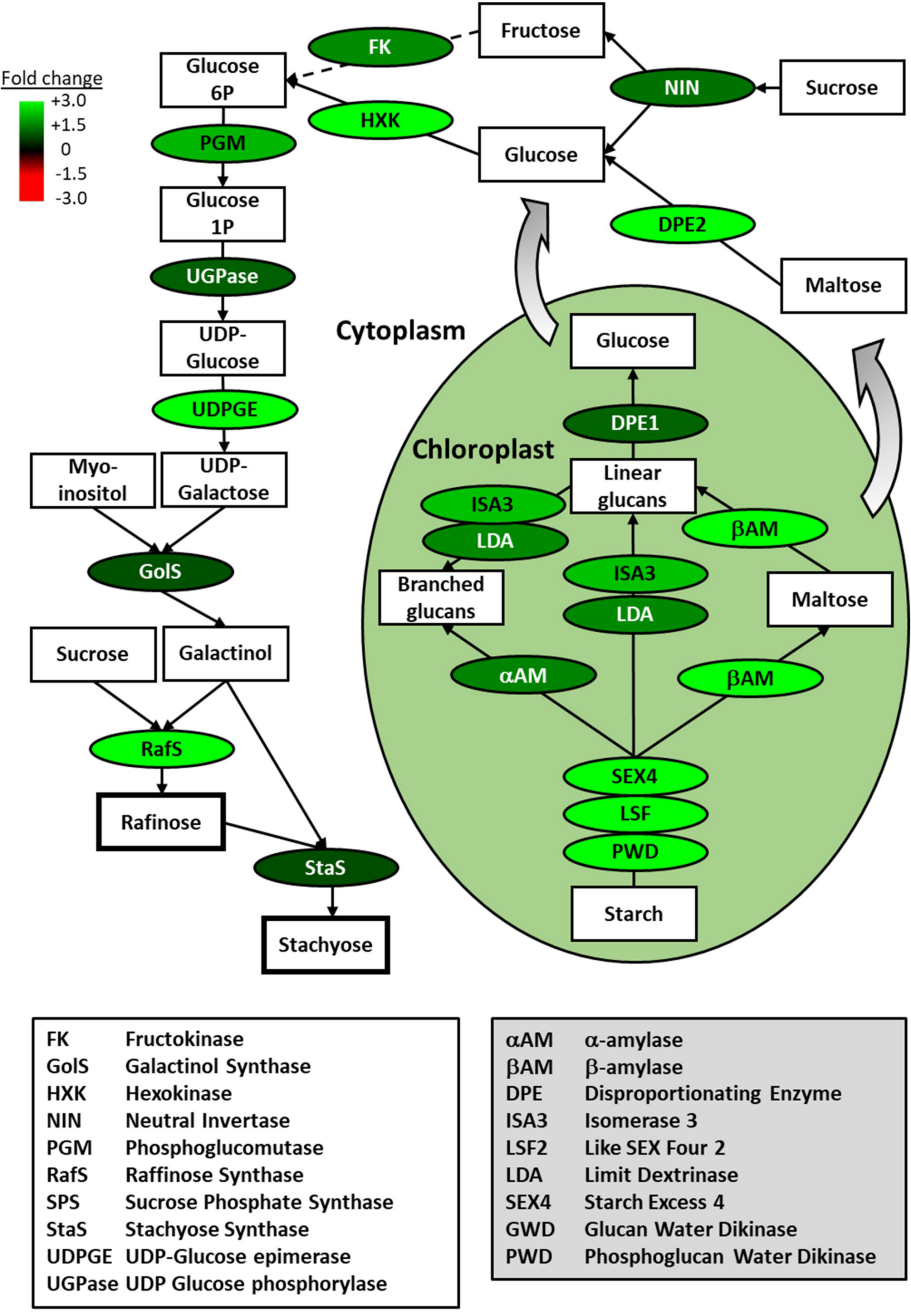
Gene expression associated with production of raffinose family oligosaccharides is strongly upregulated by end-of-production midday supplementary FR treatment. Overview of starch breakdown and soluble sugar metabolic pathways leading to raffinose family oligosaccharides showing fold change in gene expression for key enzymes in basil plants during end-of-production midday supplementary FR treatment. Enzymes are indicated in ovals and metabolic products are indicated in rectangles. Fold change in expression for genes encoding the indicated enzymes as measured by RNAseq is shown relative to untreated plants along with cytoplasmic or chloroplastic location of the enzymic reaction. Scheme based on a scheme from Tarkowski and Van den Ende (2015).

The largest functional group of genes represented by these overrepresented parent GO terms is major CHO metabolism. This overrepresentation was also supported by a more stringent GO analysis carried out by inputting the associated AGI codes of basil genes that were identified as being differentially regulated by five-fold or more during FR treatment into the Panther functional classification tool to analyze for enrichment among the GO-Slim Biological Process annotation set (Mi et al., 2019). For upregulated genes, “monosaccharide metabolic process” was enriched 9.48-fold (p = 0.0005); and its parent terms, “carbohydrate metabolic process” and “metabolic process” were enriched 4.37-fold (p = 0.00002) and 1.49-fold (p = 0.04) respectively. Given the previously-demonstrated importance of soluble sugars, particularly, raffinose and stachyose, in chilling tolerance in many species (Tarkowski and Van den Ende, 2015), we overlaid the expression patterns of genes identified within our basil transcriptome onto these metabolic pathways. Forty-five genes were identified within the major CHO metabolism functional group in our basil transcriptome, allowing the majority of enzymic steps leading from starch to raffinose and stachyose to be covered. All enzymic steps without exception showed some degree of upregulation at the level of gene expression (**Figure 7**; **Supplementary Figure 4**)

**Figure 8:**
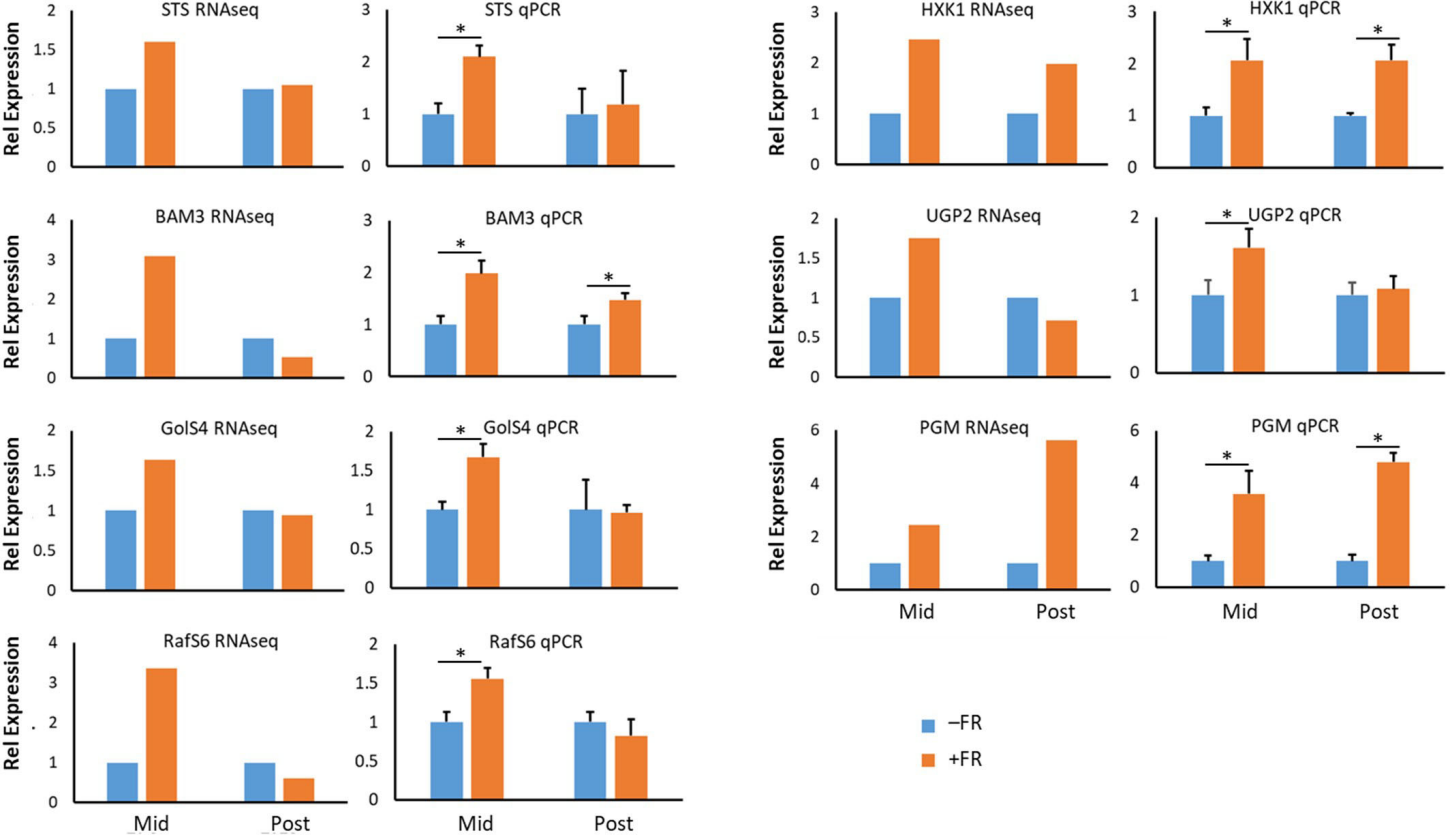
qPCR confirmation of RNAseq expression patterns in basil treated with end-of-production midday supplementary FR. Change in expression of selected genes encoding enzymes involved in starch breakdown and soluble sugar metabolic pathways leading to raffinose family oligosaccharides in basil. Expression during (Mid) and 2 h after (Post) midday supplementary FR is shown relative to untreated plants. RNAseq data is shown alongside qPCR data for each gene. qPCR data are mean ± SE for a minimum of 3 independent biological replicates. Gene symbols used are those of Arabidopsis orthologues. BAM3 – β-amylase 3, RafS6 –Raffinose synthase 6, GolS4 – Galactinol synthase 4, HXK1 – Hexokinase, PGM – Phosphoglucomutase, STS – Stachyose synthase, UGP2 – UDP-glucose pyrophosphorylase. Asterisks represent significant differences, p ≤ 0.05.

In order to confirm the patterns of gene expression observed in our RNAseq analysis, seven genes in the carbohydrate metabolism pathways represented in **Figure 7** were selected for confirmatory qPCR analysis. RNA was extracted from plants taken from three independent biological experiments replicating the treatments applied in the RNAseq analysis. In all cases mean expression patterns seen via qPCR qualitatively replicated those observed in the RNAseq analysis (**Figure 8**) and confirmed that the changes in gene expression highlighted in RNAseq data for these genes represent significant changes. The close relationship between the RNAseq data and qPCR for these sugar metabolism genes, furthermore, validates the wider patterns of gene expression associated with the induction of cold tolerance by periodic mid-day FR treatment at end of production.

## Discussion

We have demonstrated the effectiveness of four days of daily periodic 4 h mid-day supplementary FR illumination applied at end of production in triggering cold tolerance in potted basil. Basil is very susceptible to chilling injury, with short duration exposure to temperatures of even 12 °C able to cause wilting and blackening of leaves (Fratianni et al., 2017). Susceptibility of basil to chilling injury creates a major challenge for the transport of plants during winter and early spring. Conveyance from glasshouse to market requires heated conditions which adds a cost to production. Warm conditions are, furthermore, not ideal for the transport of more hardy plants, meaning that the optimum conditions for these more temperate crops are then compromised. Short term storage at the point of sale prior to display can also result in exposure to cold temperatures and result in damage causing loss of appeal to the consumer and reduction in shelf life in cold-susceptible live plants.

Daily, periodic FR illumination, applied for four days at the end of production, led to a dramatic reduction in visible signs of chilling injury in potted basil following a prolonged 24 h treatment at 4 °C. At a physiological level, periodic daily FR illumination led to a striking reduction in electrolyte leakage, an indicator of membrane damage, and greatly reduced levels of stress-associated ROS accumulation in response to cold. The treatment also mitigated the impact of cold on antioxidant levels, one of the key health benefits of basil consumption.

Chilling injury causes similar physiological changes in numerous other horticultural crops. For example, cellular damage leading to electrolyte leakage has been observed in the fruit of tomato, cucumber and eggplant (Tatsumi et al., 1981; King and Ludford, 1983; Gao et al., 2015); antioxidant loss in tomato and cucumber (Hariyadi and Parkin, 1991; Imahori et al., 2016) and accumulation of reactive oxygen species in tomato, cucumber and eggplant (Lukatkin, 2002; Zaro et al., 2014; Imahori et al., 2016). Similarly, the overt changes, wilting and discoloration of leaves, are common in ornamental plants such as poinsettia (Staby et al., 1978). The end-of-production, periodic, midday FR illumination examined here could constitute a feasible approach to induce cold tolerance in other greenhouse-grown horticultural crops such as these. Certainly, constant FR supplementation in a controlled environment growth facility has been shown to induce chilling resilience in tomato as it did in fresh cut basil (Wang et al., 2016; Larsen et al., 2023). However, the periodic midday, end-of-production, FR illumination shown to be effective here for potted basil has the advantage that it would be viable in large-scale greenhouse cultivation. Only the proportion of the crop that was at the end of production stage would need to be illuminated at any one time and only short four-hour duration treatments would be required for just four days, minimizing capital and energy costs. Produce showing chilling injury is routinely discarded for not meeting consumer expectations, resulting in considerable wastage. The majority of the 50 highest-traded fruit and vegetable commodities globally are susceptible to chilling injury which has been estimated to result in annual wastage valued at over USD 100 million (Albornoz et al., 2022). Similarly, heated transport and storage cause additional costs for both growers and distributors meaning that the approach documented here could have wide significance for the horticulture industry.

Comparative transcriptomic analyses of basil plants during and after FR treatment was used to monitor potential long term physiological changes induced by FR treatment. We identified 9,728 transcripts that showed high homology to proteins expressed in Arabidopsis, allowing us to perform a pathway level analysis of the response. In terms of pattern of response, the largest group of genes was those showing a transient response while the plants were subject to the supplementary FR light. Including both up and downregulated genes, 13.4% of the expressed genome showed a rapid transient response to shade treatment, while approximately a quarter of those genes showed prolonged regulation beyond the cessation of the supplementary FR. Immediately upon removal of FR illumination, the R:FR would return to ambient levels leading to an immediate restoration of the phytochrome photoequilibrium in favour of the Pfr form. mRNA levels of key phytochrome-responsive genes involved in shade avoidance have been shown to rapidly revert to basal levels within one hour following the removal of supplementary FR (Roig-Villanova et al., 2006), consistent with an immediate reversal of shade effects on elongation growth (Cole et al., 2011). However, clearly, a significant impact of the FR supplementation persists beyond that consistent with the observed ongoing effect observed for chilling tolerance.

The application of mid-day supplementary FR illumination was previously shown to activate the *CBF* transcription factor pathway in Arabidopsis, conferring freezing tolerance (Franklin and Whitelam, 2007). The additional involvement of the CBF pathway in chilling tolerance in a number of plant species (Thomashow, 2010) suggested that this might also provide a mechanism to improve chilling tolerance in basil. Indeed, key circadian clock components, including CIRCADIAN CLOCK ASSOCIATED 1 (CCA1) and LATE ELONGATED HYPOCOTYL (LHY), are known to regulate cold-responsive CBF pathway gene expression by acting as transcriptional activators only during the day (Kidokoro et al., 2009). In tomato, the prolonged growth of plants in monochromatic FR or in mixed R and FR light to generate a low R:FR ratio throughout the photoperiod, which has been shown to induce chilling tolerance, was demonstrated to involve FR-responsive *CBF* gene induction, also involving JA and ABA signaling (Wang et al., 2016). However, we were unable to detect any Arabidopsis CBF gene homologues in our transcriptome where the p-value of the top match was below 1x10^-4^ and the bit score above 50 (Pearson, 2013). This contrasts with the findings of Zhan et al. (2016) who identified a number of CBF response pathway genes in the related American basil, *O. americanum*, via a wider homology search. The majority of these had a low identity match to the species searched so it is possible that our pathway level analysis approach which, though necessity, used the Arabidopsis proteome as a reference, did not pick up *O. basilicum* homologues because of the greater evolutionary distance to Arabidopsis. However, the study by Zhan et al. (2016), which was aimed at characterizing the *O. americanum* response to cold, detected no change in expression of the key *COR* gene targets of the CBF pathway, consistent with basil’s poor acclimation response to cold. Our assay was carried out in a 12 h photoperiod during winter when chilling injury during transport is most likely, but we predict that the approach will also be suitable to confer chilling tolerance in longer photoperiods. The 4 h treatment in the middle of the day was given between 4 h and 8 h after dawn which was the period shown by Franklin and Whitelam (2007) to be time of maximal FR-responsiveness of the cold response pathway in Arabidopsis. Underlying that is the work of Fowler et al. (2005) and Harmer et al. (2000) who showed that, in Arabidopsis, the key cold-regulating *CBF3* gene showed a peak of circadian expression at this time in a 12 h photoperiod. We checked publicly available Arabidopsis diurnal gene expression datasets available via the “Diurnal” web tool (Mockler et al., 2007) and confirmed that the *CBF3* gene continue to cycle in both long (16 h) and short (8 h) photoperiods (**Supplementary Figure 5**), therefore, supporting the proposal that periodic daytime FR supplementation will also be effective in these photoperiods. Crucially, however, the peak of *CBF3* gene expression in Arabidopsis remained consistent at 8 h after dawn in these datasets irrespective of the photoperiod suggesting that this same period between 4 h and 8 h after dawn may also be the key time for application of supplemental FR in order to induce cold tolerance in longer or shorter photoperiods. Our assay used FR supplementation which achieved an R:FR of 0.16, consistent with that used by Franklin and Whitelam (2007). It would also be interesting to determine whether a less extreme low R:FR ratio could also confer cold tolerance in basil. Any reduction in R:FR ratio below 0.8 has been demonstrated to be effective in inducing shade avoidance (Smith, 1982). Consistent with this, Wang et al. (2016) demonstrated that chilling tolerance in tomato could also be triggered by growth in light with a R:FR of 0.5. It will be possible to investigate in the future whether less intense periodic midday supplementary FR illumination would also be effective in inducing chilling tolerance in basil as this would make the treatment even more cost-effective for a commercial setting.

Pathway analysis based on our transcriptomic data showed that FR supplementation triggered a downregulation of photosynthesis-related gene expression during illumination. Acclimation in photosynthetic processes is classically associated with the shade avoidance response (Casal, 2012). However, 2h after the cessation of FR supplementation, we no longer observed any enrichment of photosynthetic processes among downregulated genes, suggesting that there would not be any long-term detriment of the 4 h FR supplementation treatment in terms of photosynthate accumulation. Our assay of ROS levels, however, may reflect an additional effect of supplementary FR on the photosynthetic machinery. We observed a small increase in H_2_O_2_ in leaves in FR-treated plants. Some of the additional wavelengths in the 700 –800 nm range would be absorbed by the photosystems (Pettai et al., 2005) and it may be that the higher level of light absorption caused the formation of some additional ROS due to photo-oxidative stress. A transient increase in TCA cycle-related gene expression during supplementary FR illumination was also observed in our pathway analysis. This also concurs with previous metabolite analysis in Arabidopsis which revealed higher levels of TCA intermediates in response to shade (Yang et al., 2016; Krahmer et al., 2021). However, again, this alteration was not observed to persist in our assay 2 h beyond the removal of the FR illumination.

Interestingly most other pathways showing enrichment in our analysis showed persistent changes in gene expression. The most dramatic enrichment was seen among upregulated genes associated with major carbohydrate metabolism. The shade avoidance response is known to cause significant changes in metabolism and recent metabolic analyses have also shown that phytochrome modulates the balance between starch and sugar metabolism in Arabidopsis Krahmer et al., 2021). This is particularly significant given the key involvement of alteration of sugar metabolism in the cold acclimation response (Tarkowski and Van den Ende, 2015). Indeed, this is known to be one of the key downstream targets of CBF signaling in Arabidopsis (Tarkowski and Van den Ende, 2015). Notably, despite not seeing any alteration in expression of CBF pathway components in American basil in response to cold, Zhan et al. (2016) did find an over-representation of genes associated with starch and sucrose metabolism among genes differentially regulated in response to cold in *O. americanum* and these were proposed to be involved in chilling tolerance. Similarly, Larsen et al. (2023) in their assay applying supplementary FR to basil throughout the photoperiod for one or three weeks prior to harvest in a vertical farm set-up, demonstrated observed increased levels of soluble sugars and proposed that this could be central to the acquisition of chilling tolerance.

Monosaccharides, generally, improve osmotic protection but raffinose family oligosaccharides (RFOs) have particularly been implicated in providing tolerance to chilling, potentially protecting membrane stability, particularly of the chloroplast membranes (Nägele and Heyer, 2013; Tarkowski and Van den Ende, 2015) and also scavenging reactive oxygen species (Couée et al., 2006). Phytochrome has previously been shown to influence synthesis of rafinose (Yang et al., 2016; Krahmer et al., 2021) and, in our assay, all transcripts coding for enzymes involved in the pathway from starch utilization through to synthesis of the key RFOs, the trisaccharide raffinose, and the tetrasaccharide stachyose, were upregulated in in response to FR supplementation (**Figure 7**; **Supplementary Figure 4**). This included expression of the gene encoding galactinol synthase, the first committed and rate limiting step in RFO production. Although the upregulation of expression was not persistent beyond FR treatment for all of the specific RFO enzymes (unlike sugar metabolism more generally), it is expected that resultant enzyme levels and effects on RFO sugars would persist for some time beyond the FR treatment. Overexpression of galactinol synthase has been demonstrated to confer increased stress resistance in Arabidopsis and in chickpea (Taji et al., 2002; Salvi et al., 2018) and, more specifically, chilling tolerance in rice (Shimosaka and Ozawa, 2015), while metabolites of the raffinose pathway were also shown to accumulate as part of cold acclimation in strawberry (Koehler et al., 2015). A persistent downregulation of transcripts associated with cell wall metabolism was also observed in response to supplementary FR. Some links between cell wall metabolism and cold tolerance have recently been suggested (Xu et al., 2020) but little is known in terms of any mechanistic links. However, phytochrome is known to balance the allocation of resources between growth and resilience (Devlin, 2016) meaning this could represent a simple reallocation of resources, particularly carbohydrates, from cell wall production towards RFO production.

Alterations in fatty acids have also commonly been associated with cold acclimation, with desaturation of membrane fatty acids reducing the tight packing of membrane lipids and counteracting the decreased membrane fluidity associated with cold temperatures (Ruelland et al., 2009). Our transcriptomic analysis revealed persistent downregulation of fatty acid metabolism as a whole but particularly, we observed an enrichment of the ontology term, fatty acid desaturation, among genes downregulated following FR supplementation. Similarly, alterations in levels amino acids are also associated with improved cold tolerance. For example, proline, in particular, is known to act as an osmoprotectant to help stabilize proteins (Ruelland et al., 2009). Although our GO term enrichment analysis did not identify any enrichment of genes involved in proline biosynthesis among differentially-regulated genes, terms associated with BCAA synthesis, and serine-glycine-cysteine group synthesis were overrepresented among FR upregulated genes while aromatic amino acid synthesis was overrepresented among FR downregulated genes. Mass spectrometric analysis of cold acclimation in strawberry (Koehler et al., 2015) also found that the BCAA branch of amino acid biosynthesis, leading from pyruvate to the structures of isoleucine, leucine, and valine, was upregulated. The authors suggest that, while BCAAs are not generally associated with cold response, they are possibly also of general osmotic protective value in the same way as proline. Accumulation of serine has also previously been observed as part of a cold acclimation response in *Lolium perenne* (Bocian et al., 2015).

The observed downregulation of aromatic amino acid metabolism may be associated with a general downregulation of specialized metabolism. Shikimate pathway derived amino acids tyrosine and phenylalanine are the building blocks of phenylpropanoid biosynthetic pathways. Genes encoding phenylpropanoid and biosynthetic enzymes are strongly enriched among persistently downregulated genes. Such pathways are important in synthesis of key basil phenylpropanoid volatiles such as methyl chavicol and eugenol and the valuable polyphenol antioxidant, rosmarinic acid. Again, this concurs with the aforementioned mass spectrometric analysis of cold acclimation in strawberry which showed phenylpropanoid levels decreased in roots in response to cold in strawberry (Koehler et al., 2015). Likewise, isoprenoid metabolism also appears to be downregulated. Despite the downregulation of phenylpropanoid metabolism, there is an enrichment of genes associated with flavonoid and anthocyanin metabolism among genes upregulated after FR treatment. Anthocyanins are generally associated with cold tolerance in plants (Janská et al., 2010) and a mass spectrometric analysis in Arabidopsis identified an upregulation in anthocyanins as a key part of cold acclimation (Schulz et al., 2016). There are also both short term and persistent effects of supplementary FR on isoprenoid metabolism. The non-mevalonate (non-MVA) or methyl-D-erythritol phosphate (MEP) pathway, comprising early steps in the isoprenoid pathway is enriched among short term downregulated genes but not among persistently FR-downregulated genes suggesting it likely returns to normal following the cessation of the FR supplementation. This pathway contributes to the synthesis of key isoprenoid building blocks that got on to form a wide range of key plant molecules such as terpenoids, carotenoids and gibberellins. However, later steps of the terpenoid pathway, which include terpene synthases, show longer term downregulation suggesting that synthesis of key basil terpene volatiles such as linalool and 1,8-cineole may be downregulated. Again, this may represent a reallocation of resources from specialized metabolism towards resilience.

There was also a persistent effect of FR supplementation on the expression of genes in a number of hormone biosynthesis pathways. GO terms associated with brassinosteroid metabolism were overrepresented among persistently downregulated genes while GA and JA metabolism terms are overrepresented among upregulated genes. The FR-induction of chilling tolerance in tomato observed by Wang et al. (2016) was found to be dependent on ABA and JA signaling. The indicated upregulation of JA metabolism in our study suggests that there may be a parallel in basil in terms of JA involvement in FR-induced chilling tolerance. However, no enrichment of genes involved in ABA biosynthesis was observed among our differentially-regulated genes and this agrees with measurements of ABA carried out by Larsen et al. (2023) following growth of basil in supplementary FR for one or three weeks. Conversely, though, Larsen et al. (2023) also saw no change in JA levels in basil under these conditions raising the possibility that changes in actual levels of these hormone may be subtle if they are involved in FR-induced cold acclimation in basil. GAs have previously been shown to be a key part of cold acclimation in plants, with the accumulation of protective sugars having been shown to be downstream of reduced GA levels (Tarkowski and Van den Ende, 2015). This downregulation of GAs in cold acclimation is mediated via the upregulation of specific GA-deactivating GA-2-oxidase (*GA2OX*) genes (Colebrook et al., 2014). Indeed, homologues of gibberellin 2-oxidase 2 (*GA2OX2*) and gibberellin 2-oxidase 1 (*GA2OX1*) were both strongly upregulated in a persistent manner in our assay (**Supplementary Table 2**).

The GO term, brassinosteroid metabolism, was enriched among persistently downregulated genes following FR. Brassinosteroids are derived from sterols (Andrzej et al., 2020) and a closer inspection of the specific downregulated genes tagged in this pathway revealed that this enrichment actually reflects a downregulation of sterol metabolism, with the homologues of squalene epoxidase 1 (*SQE1*), *SQE2* and *SQE3*all showing substantial downregulation (**Supplementary Table 2**). Homologues of cycloeucalenol cycloisomerase (*CPI1*) and cycloartenol synthase 1 (*CAS1*), the enzymes catalyzing the later, first committed steps of brassinosteroid synthesis (Du et al., 2022) showed little or no response to supplementary FR. Sterols are associated with increased membrane fluidity and so, generally, decreased sterols are associated with reduced cold tolerance (Du et al., 2022). For example, a reduction in sterol content aggravated the cold stress injury of wheat (Valitova et al., 2019). However, the squalene epoxidases of the sterol biosynthetic pathway are also a key part of the pathway of triterpene synthesis (Olofsson et al., 2011) so this downregulation may be associated with reduced synthesis of triterpenes such as oleanolic acid in basil as part of the wider downregulation of specialized metabolism including essential oil and phenylpropanoid metabolism as would be expected associated with the reallocation of resources towards resilience seen in response to supplementary FR (Devlin, 2016).

### Summary

Our study, building upon previous work indicating that induction of cold response pathways by FR is gated by the circadian clock (Fowler et al., 2005; Franklin and Whitelam, 2007), demonstrated that 4 h of supplementary FR given around midday in a 12 h photoperiod for only 4 days prior to end of production was able to induce cold tolerance in potted basil. The treatment was able to confer tolerance to 24 h of 4 °C temperature exposure in living plants, sufficient for transport of pots to market and short-term storage at a retailer after unloading. The method offers a non-invasive approach to induce chilling tolerance which is suitable for application in a large-scale commercial glasshouse, requiring only 4 days of end-of-production treatment and conferring cold tolerance without induction of shade avoidance responses outside of the 4 h treatment window. The short duration of treatment also makes this a relatively energy efficient treatment. The treatment significantly reduced the occurrence of visible chilling damage including wilting and discoloration while also dramatically reducing physiological markers of chilling damage such as electrolyte leakage and reactive oxygen species accumulation. Benefits were also demonstrated in terms of reducing the loss of antioxidant levels.

**Figure 9:**
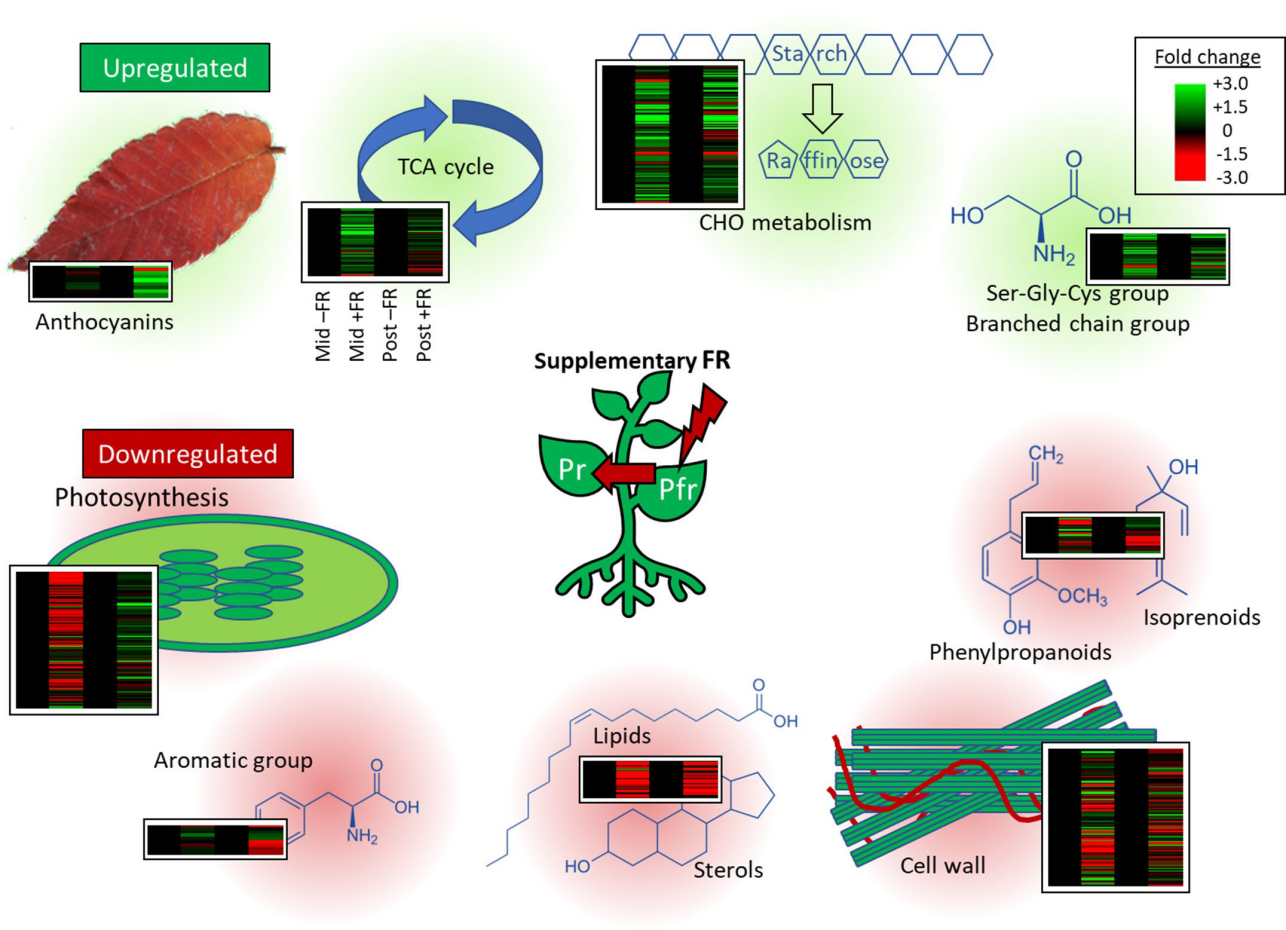
Expression of individual transcripts in key pathways differentially regulated by end-of-production midday supplementary FR. Heatmaps show RNAseq expression patterns of all transcripts within significantly enriched MapMan gene ontology categories identified among up- or downregulated genes. For each heatmap, columns represent expression in FR-treated (+FR, columns 1 and 3) relative to untreated (–FR, columns 2 and 4) plants at the same timepoint, either during (Mid, columns 1 and 2) or 2 h after (Post, columns 3 and 4) supplementary FR treatment. Each transcript is represented by a separate line. Central figure represents the effect of supplementary FR on phytochrome photoequilibrium. Gene ontology categories enriched among upregulated genes are shaded in green. Gene ontology categories enriched among downregulated genes are shaded in red.

Transcriptomic analysis revealed persistent increases in expression of genes involved in a number of pathways widely associated with cold tolerance. Upregulation was observed for genes involved in major carbohydrate metabolism and, in particular, the synthesis of RFO sugars; genes in pathways associated with the biosynthesis of branched chain and serine-glycine-cysteine group amino acids; and genes involved in anthocyanin synthesis, all of which have previously been proposed to have protective roles in cold stress (**Figure 9**). Persistent downregulation was observed for genes in pathways associated with growth and, in particular, cell wall metabolism, and genes associated with specialist metabolism, in particular, phenylpropanoids and isoprenoids, potentially reflecting the commonly observed trade-off between growth and resilience associated with acclimation to stress. A short-term downregulation of genes associated with photosynthesis and upregulation of genes associated with the TCA cycle was also observed during supplementary FR treatment but these did not persist beyond the 4 h treatment period. Given that susceptibility to chilling injury creates a major challenge for the transport of many live herbaceous plants during winter and early spring, it will be interesting to determine whether the approach has general applicability to other cold sensitive species.

## Data availability statement

The data presented in the study are deposited in the NCBI SRA repository, accession number PRJNA995591.

## Author contributions

FB, GS and SS contributed to experimental design and carried out the experiments. SB and AS contributed to experimental design and supervised the project. PD conceived the original idea, contributed to experimental design, supervised the project and wrote the bulk of the manuscript. All authors discussed the results and contributed to the final manuscript.
