# Supplementary material for: Improved chilling tolerance in glasshouse-grown potted sweet basil by end-of-production, short-duration supplementary far red light
Source: Front Plant Sci. 2023 Aug 16;14:1239010. doi: 10.3389/fpls.2023.1239010 (PMC10468977; doi:10.3389/fpls.2023.1239010)
Supplement: Supplementary file 1 [file DataSheet_1.docx]

Supplementary Material

Improved chilling tolerance in glasshouse-grown potted sweet basil by end-of-production, short-duration supplementary far red light

Firdous U. Begum, George Skinner, Sandra P. Smieszek, Simon Budge, Tony D. Stead, Paul F. Devlin^*^

*** Correspondence:** Paul F Devlin: Paul.devlin@rhul.ac.uk

# Supplementary Figures and Tables

## Supplementary Figures


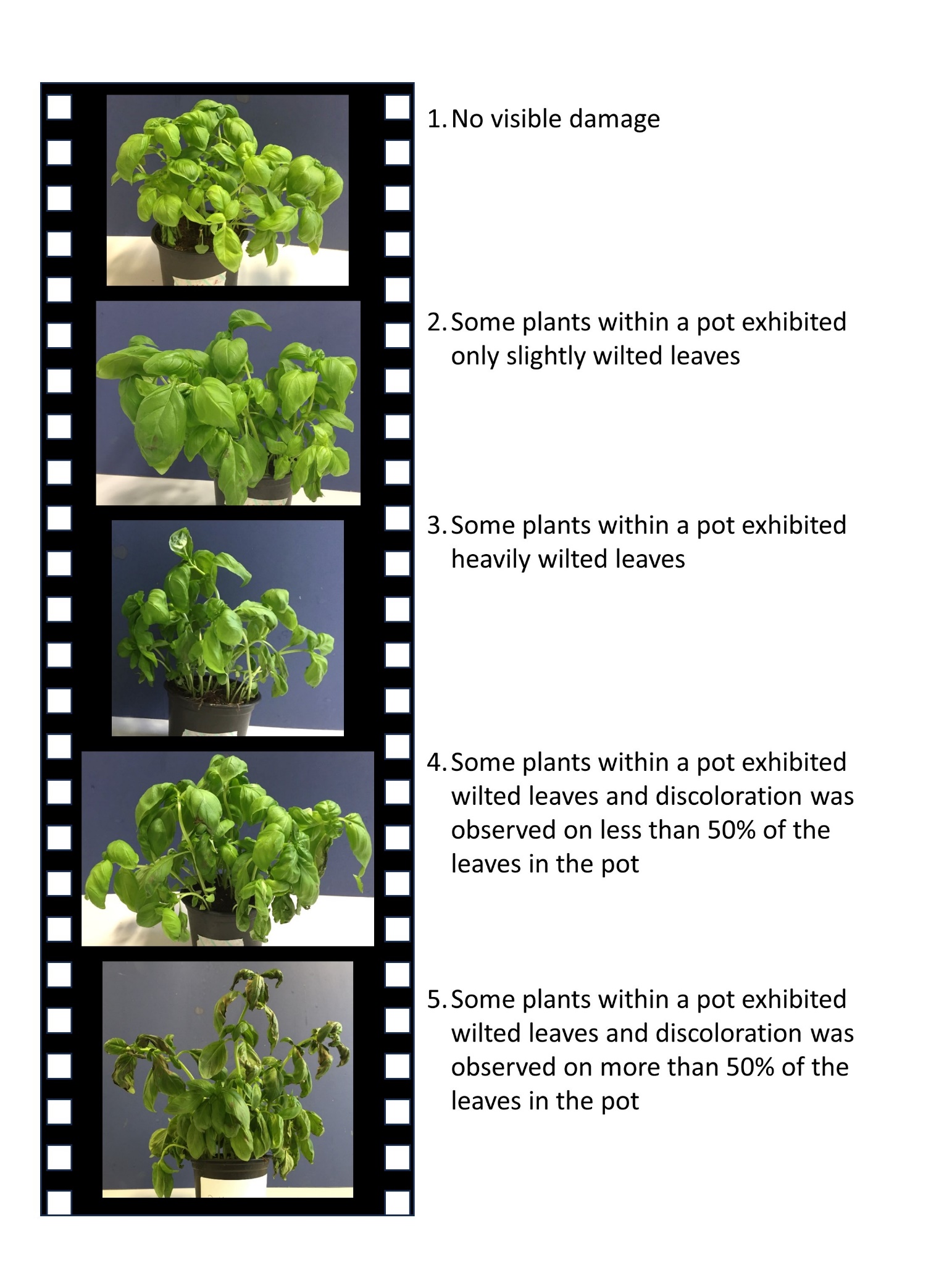


**Supplementary Figure 1.** Representative pots illustrating the chilling injury scale used in assessing of visible damage after 24 h incubation at 4 ^o^C. Individual pots were assigned a number as indicated.

**A**


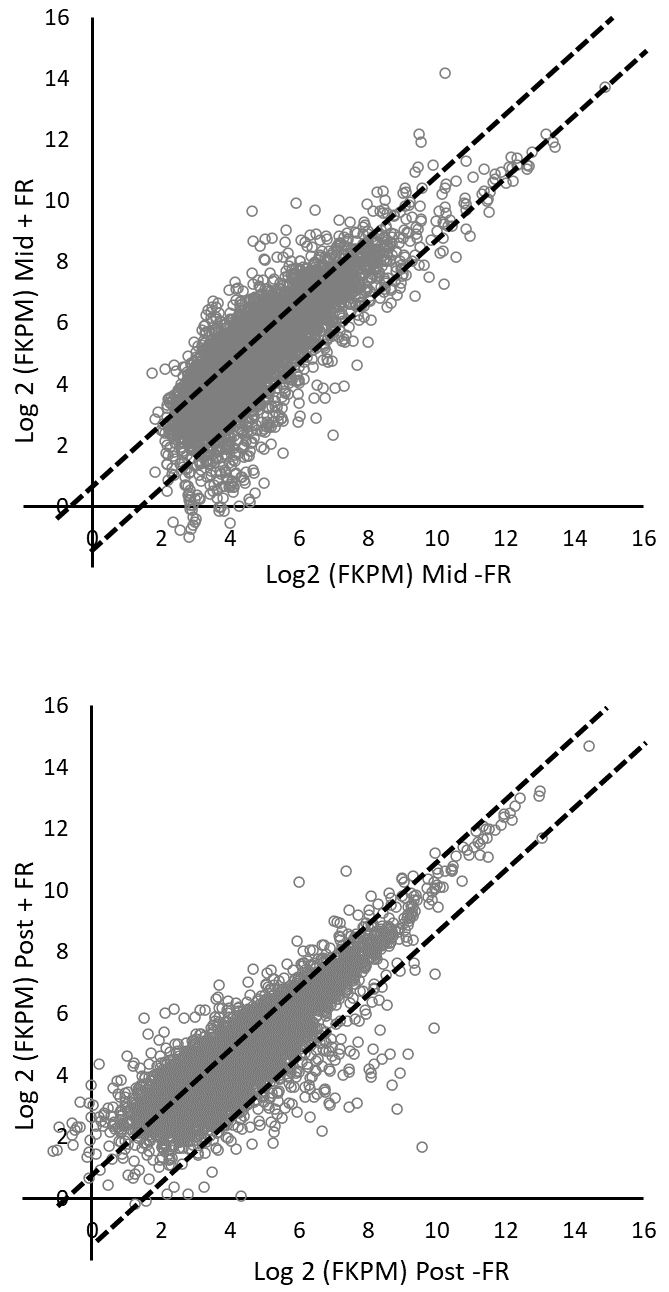


**B**

**Supplementary Figure 2.** Global transcript abundances in control versus FR treated plants during (A) and 2 h after (B) supplementary FR treatment. Each point represents a distinct transcript. Transcripts outside of the dotted lines show a two-fold difference in expression compared to control.

**
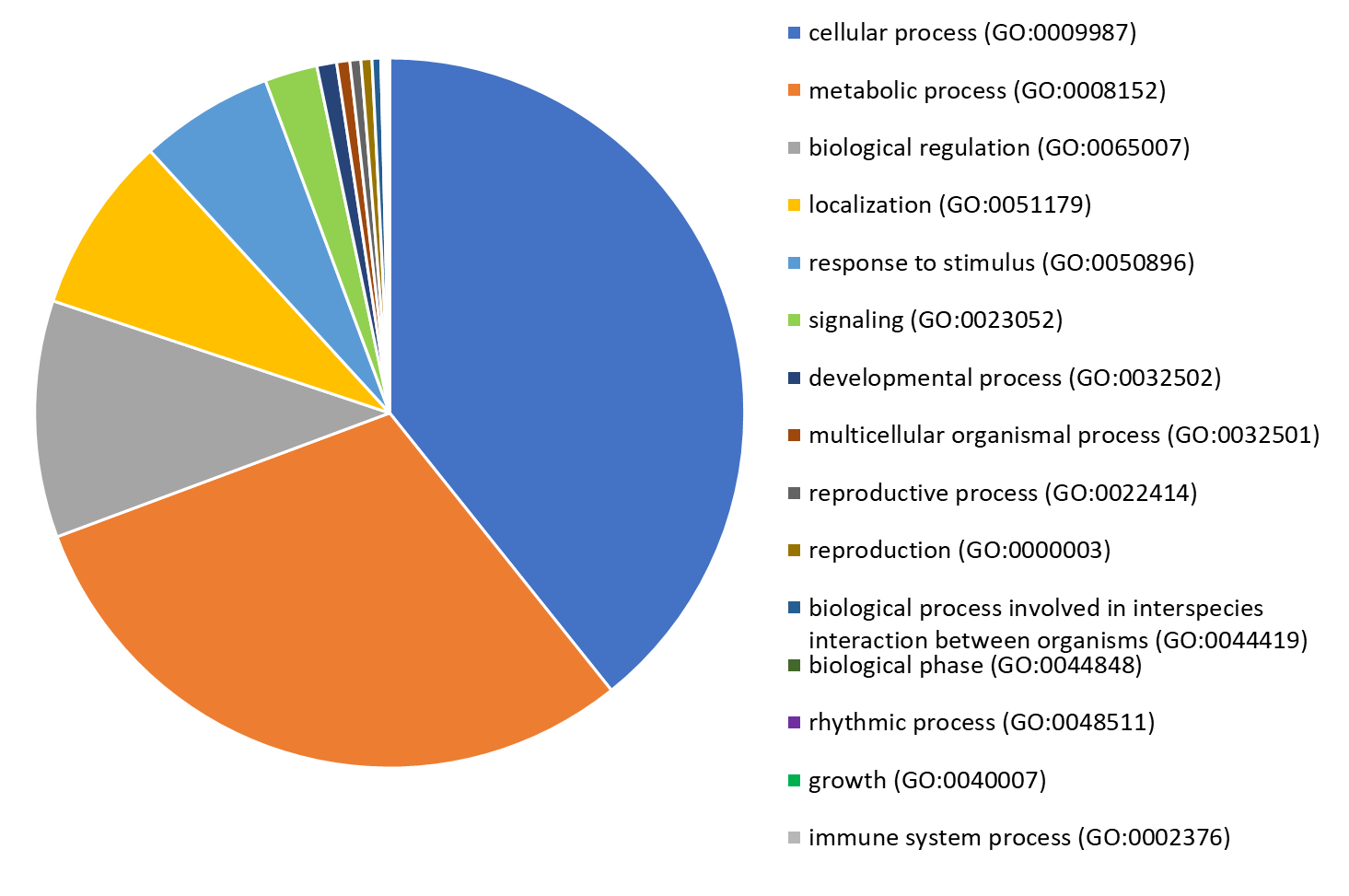
**

**Supplementary Figure 3.** Superficial genome-wide gene ontology (GO) analysis cataloging biological processes for all basil transcripts that were mapped to Arabidopsis orthologues.


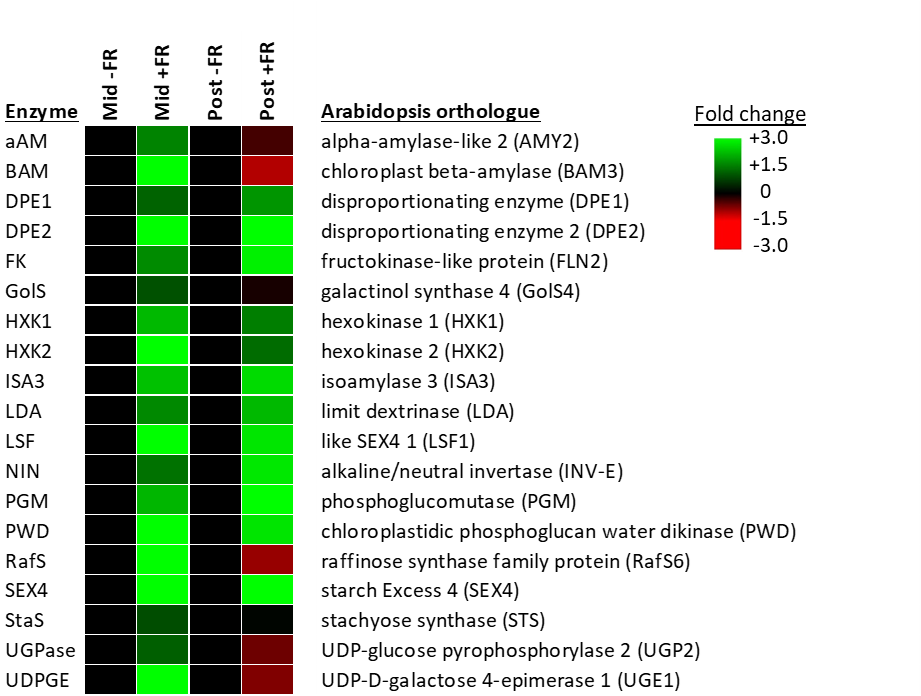


**Supplementary Figure 4.** Relative expression of genes associated to the GO category, Major CHO Metabolism in control versus FR treated plants during (Mid) and 2 h after (Post) supplementary FR treatment. Enzyme name abbreviations correspond to those in Figure 7 alongside the Arabidopsis orthologues identified for each enzyme in the basil transcriptome.

**
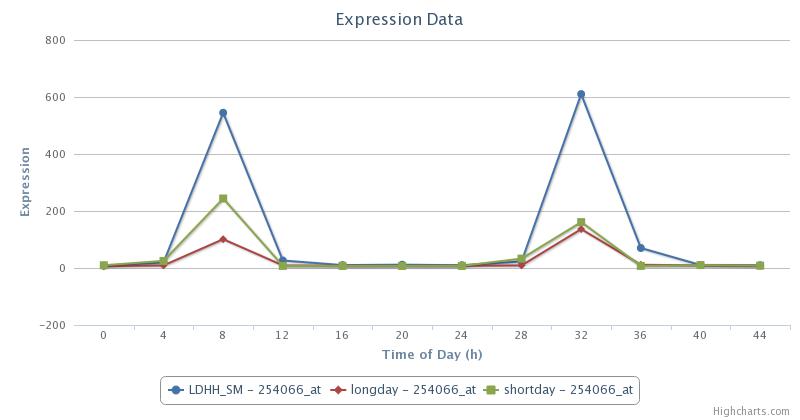
Supplementary Figure 5.** Relative expression of the *CBF3* gene in Arabidopsis in 12 h- (LDHH_SM), 16 h- (longday) and 8 h- (shortday) photoperiods. Figure downloaded from the publicly available Arabidopsis diurnal microarray gene expression database, “Diurnal”, (<http://diurnal.mocklerlab.org/>). Time of day is measured as hours since dawn on the first of the two days shown.

## Supplementary Tables

**Supplementary Table 1.** Primers used for qRT-PCR confirmation of gene expression.

| *A. thaliana* orthologue | Gene symbol | Basil primer | Sequence |
| --- | --- | --- | --- |
| AT4G17090 | BAM3 | Ob-BAM3-F | TGGATTGGCGTTCTTCCTCT |
|  |  | Ob-BAM3-R | GTCGACGGAACCCAGAGTAT |
| AT5G20250 | RafS6 | Ob-RafS6-F | CCGCCCAAATTCGTCATCAT |
|  |  | Ob-RafS6-R | GTTAGTCTCTGCAGCTTGGC |
| AT1G60470 | GolS4 | Ob-GolS4-F | ACACTGGGGAGGAAGCTAAC |
|  |  | Ob-GolS4-R | CCCACCATTTCTTCACCAGC |
| AT4G29130 | HXK1 | Ob-HXK1-F | GGACAAGATGTGGTGGGAGA |
|  |  | Ob-HXK1-R | ACGTGCATATCAAGGCCAAC |
| AT1G70820 | PGM | Ob-PGM-F | GAGAAAGGGAGGACGGTGAA |
|  |  | Ob-PGM-R | TTGGGTCCCTTCCTAACGAC |
| AT4G01970 | STS | Ob-STS-F | AACACAACCTCCCCTCCATT |
|  |  | Ob-STS-R | TCAACAGTGGAGGGACGATC |
| AT5G17310 | UGP2 | Ob-UGP2-F | AGCTCAACATGTCGAGTGGA |
|  |  | Ob-UGP2-R | TTTGGTCTCAGCGGGATCTT |
| n/a | UBQ | Ob-UBQ-F | ATTGGAGGTGGAGAGTTCGG |
|  |  | Ob-UBQ-R | CCTTCCATCCTCCAACTGCT |
